# A two-stage dominance-based surrogate-assisted evolution algorithm for high-dimensional expensive multi-objective optimization

**DOI:** 10.1038/s41598-023-40019-6

**Published:** 2023-08-13

**Authors:** Mengjiao Yu, Zheng Wang, Rui Dai, Zhongkui Chen, Qianlin Ye, Wanliang Wang

**Affiliations:** 1 College of Computer Science and Technology, Zhejiang University of Technology, Hangzhou, 310015 China; 2 School of Computer and Computational Sciences, Zhejiang University City College, Hangzhou, 310015 China

**Keywords:** Engineering, Mathematics and computing

## Abstract

In the past decades, surrogate-assisted evolutionary algorithms (SAEAs) have become one of the most popular methods to solve expensive multi-objective optimization problems (EMOPs). However, most existing methods focus on low-dimensional EMOPs because a large number of training samples are required to build accurate surrogate models, which is unrealistic for high-dimensional EMOPs. Therefore, this paper develops a two-stage dominance-based surrogate-assisted evolution algorithm (TSDEA) for high-dimensional EMOPs which utilizes the RBF model to approximate each objective function. First, a two-stage selection strategy is applied to select individuals for re-evaluation. Then considering the training time of the model, proposing a novel archive updating strategy to limit the number of individuals for updating. Experimental results show that the proposed algorithm has promising performance and computational efficiency compared to the state-of-the-art five SAEAs.

## Introduction

Multi-objective optimization problems (MOPs)^1^ are very common in real-world problems such as electrical engineering^2^, logistics scheduling^3^, and robotics^4^, which often have two or more objectives that need to be optimized simultaneously. The mathematical form of the MOPs can be expressed as follows:

1 $$ \begin{array}{*{20}c} {{\text{minimize}}\;\left\{ {f_{1} \left( x \right),f_{2} \left( x \right), \ldots ,f_{k} \left( x \right)} \right\}} \\ {subject\;to\;x \in X,} \\ \end{array} $$

where *X* is the search space of decision variables, *x* is the decision vector with *D* decision variables, and *f*_*1*_(*x*),…,*f*_*k*_(*x*) are *k* (≥ 2) objective functions to be optimized^5^. Usually, due to the conflict between objectives, it is difficult to obtain a single value that can satisfy all objectives, so we can only coordinate among multiple objectives to make them as optimal as possible^6^. The solution obtained by MOPs is not unique, but an optimal solution set composed of a set of optimal solutions, namely Pareto set (PS) in decision space and Pareto front (PF) in the objective space^7^.

Multi-objective evolutionary algorithms (MOEAs) have been popular in solving MOPs due to numerous advantages^8,9^. For instance, MOEAs can efficiently obtain a set of optimal solutions in a single run even in the presence of irregular or noisy fitness functions. Various MOEAs have been developed, which can be roughly divided into three categories. The first category is Pareto or dominance-based MOEAs^10^. For example, NSGAIII^11^ used the dominant relationship as a criterion for selection. The second includes indicator-based MOEAs^12^, such as HV^13^ adopted in HypE^14^ as the indicator. The third category is decomposition-based approaches^15^, which apply a series of vectors to decompose the MOP into multiple single-objective problems. For instance, MOEA/D^16^ and RVEA^17^ solved MOPs based on decomposition and reference vectors respectively.

It is worth noting that the majority of MOEAs typically involve more than 10,000 function evaluations (FEs) in the process of optimizations. But for some expensive multi-objective optimization problems (EMOPs)^18,19^ (such as computational electromagnetics^20^, fluid dynamics^21^, and finite element analysis^22^) whose evaluation might take hours or even days. In such cases, the time consumption and computational cost can be burdensome. Therefore, how to reduce the computational cost or the number of evaluations for EMOPs is a significant concern.

Surrogate-assisted evolutionary algorithms (SAEAs)^23^ are a representative method to solve EMOPs^18^. The surrogate model (also known as meta-model) is built by machine learning to replace the computationally expensive real fitness function, enabling the evaluation of individuals at a lower cost during each iteration^24^. A variety of machine learning methods, including the Kriging model^25^, radial basis function (RBF)^26^, and support vector machines (SVM)^27^, have been introduced as surrogate models in SAEAs. On MOPs, SAEAs also have demonstrated their effectiveness in significantly reducing the number of expensive FEs^28^. According to the modeling method, SAEAs can be roughly categorized into approximation-based and classification-based, which can be summarized below:

Approximation-based SAEAs: In the field of evolutionary computing, fitness approximation can employ various strategies, e.g. approximate fitness value or Pareto rank^29^. Among these SAEAs, the most typical ones are the Kriging and RBF^30^. In AB-MOEA^31^, an adaptive Bayesian method was proposed to determine which candidate solutions require evaluation. In K-RVEA^32^, a Kriging-assisted RVEA used the approximation ability of the Kriging model to assist optimization in selecting individuals. Besides, several machine learning methods are employed in surrogate modeling. For example, EDN-ARMOEA^33,34^ utilized a dropout neural network to achieve a good balance between convergence and diversity.

Classification-based SAEAs: The classification-based surrogate model does not evaluate individuals based on their approximate fitness values, but compares the individuals by classifiers^35^. As a classifier, the surrogate model compares the quality between the reference solution and other individuals and assigns a label to facilitate subsequent selection^36^. CSEA^37^ constructed an artificial neural network to predict the dominant relationship between the candidate solution and the reference solution. And CPS-MOEA^38^ classified the candidate solutions into positive and negative data sets by a pre-selection method.

Although the application of surrogate models is widespread, numerous challenges still remain. First, which surrogate model to choose^39^. At present, mainstream surrogate models include Kriging, RBF, SVM, etc. But there is no standard in place to determine which surrogate model should be used. The second is how to use the surrogate model^40,41^. As mentioned above, the surrogate model can be applied to approximate the fitness value or estimate the rank of the individual. The way in which surrogate models are employed greatly influence the final result. Last but not least, how to update the surrogate (i.e. selection of individuals for re-evaluation) is also an important issue. Various approaches are described in the literature, e.g. selecting a set of optimal solutions^42^, non-dominant solutions^43^, or reference solutions based on the predicted results of the surrogate model^44^.

Furthermore, SAEAs proposed in recent years have primarily focused on low-dimensional EMOPs with less than 30 decision variables. This limitation is primarily attributed to the scarcity of training samples for building accurate surrogate models and the excessive time required to train high-dimensional models. However, the critical problem of the number of decision variables has gained increasing attention. There are two important factors to consider when using the surrogate model to solve high-dimensional EMOPs. First, how selecting and building a surrogate model can minimize the extra computational cost caused by dimension increase. The efficiency of establishing and updating the surrogate model is strongly related to the dimension of the problem. Taking K-RVEA as an example, this algorithm performed well in scenarios with 10 decision variables, but its performance deteriorated significantly when the decision variable increased under the limited FEs. The second is how to find individuals with a balance of diversity and convergence. In SAEAs, such individuals are not only used for re-evaluation, but also play a crucial role in improving the accuracy of the surrogate model.

Inspired by the aforementioned concepts, we propose a two-stage dominance-based surrogate-assisted evolution algorithm (TSDEA) for high-dimensional expensive multi-objective optimization. In the algorithm, the RBF model is employed to replace a portion of the FEs and guide individual evolution. The proposed algorithm designs a reference vector based two-stage selection strategy, which convergence considers the diversity and convergence of the population during the selection process. Besides, we develop a novel archive updating strategy to limit the number of update samples for retraining the RBF model. during the selection process, the reference vector assumes a crucial role an indispensable part.

The main contributions of this paper can be summarized as follows:

1. To maintain the convergence and diversity within the population, a two-stage selection strategy is proposed. In the two-stage selection strategy, individuals are selected using the dominant relationship and angle-penalized distance (APD) proposed in RVEA, respectively. By employing this approach, we can thoroughly explore both the decision space and objective space, leading to the selection of individuals with exceptional convergence and diversity.
2. To limit the computing time of model training, an archive updating strategy is designed in this paper to make the maximum number of samples of model training limited. If the total number of samples exceeds, redundant individuals will be removed from the archive. This approach ensures that only the most relevant and informative samples are retained, optimizing the efficiency of the model training process.

The rest of the paper is organized as follows. In Section “Preliminary”, we provide a relatively brief description of the non-dominant sorting method, APD, and RBF models so that the paper is self-contained. Section “Proposed algorithm” gives a detailed description of the proposed algorithm. The experimental results and analysis are given in Section “Numerical experiments”. Finally, we draw a conclusion and prospect in Section “Conclusions”.

## Preliminary

In this section, we begin by presenting NSGAIII and angle-penalized distance (APD), which serve as the prototype and foundation of the two-stage selection strategy in this paper. Subsequently, we briefly describe the RBF model, including a comparison of other alternative models.

### NSGAIII

The TSDEA proposed builds upon the concept of non-dominant sorting in NSGAIII and incorporates some improvements. a detailed explanation of the NSGAIII algorithm is provided in this part.

In NSGAIII, a set of structured reference points is initially constructed. Next, NSGAIII continues to iterate through the operations of genetic, recombination, evaluation, non-dominant sorting, and selection until the maximum iterations are reached. At the *t*-th generation, the merger of the current population (population size is *N*) and its offspring (population size is *N*) will form a new population *P*_*t*_. *N* individuals need to be selected in the population *P*_*t*_. *P*_*t*_ is initially into several non-dominant layers (*F*_*1*_, *F*_*2*_, …, *F*_*n*_) based on the non-dominant sorting. Individuals are then selected from *F*_*i*_ to join a new population (denoted as *S*_*t*_) until the number of selected individuals reaches or exceeds *N* for the first time, and this critical layer is denoted as *F*_*L*_. If the number of individuals in *S*_*t*_ exceeds *N*, individuals in *F*_*1*_ − *F*_*L−1*_ are accommodated in *P*_*t*+*1*_, followed by a niche operation to select the remaining individuals from *F*_*L*_.

**Figure 1:**
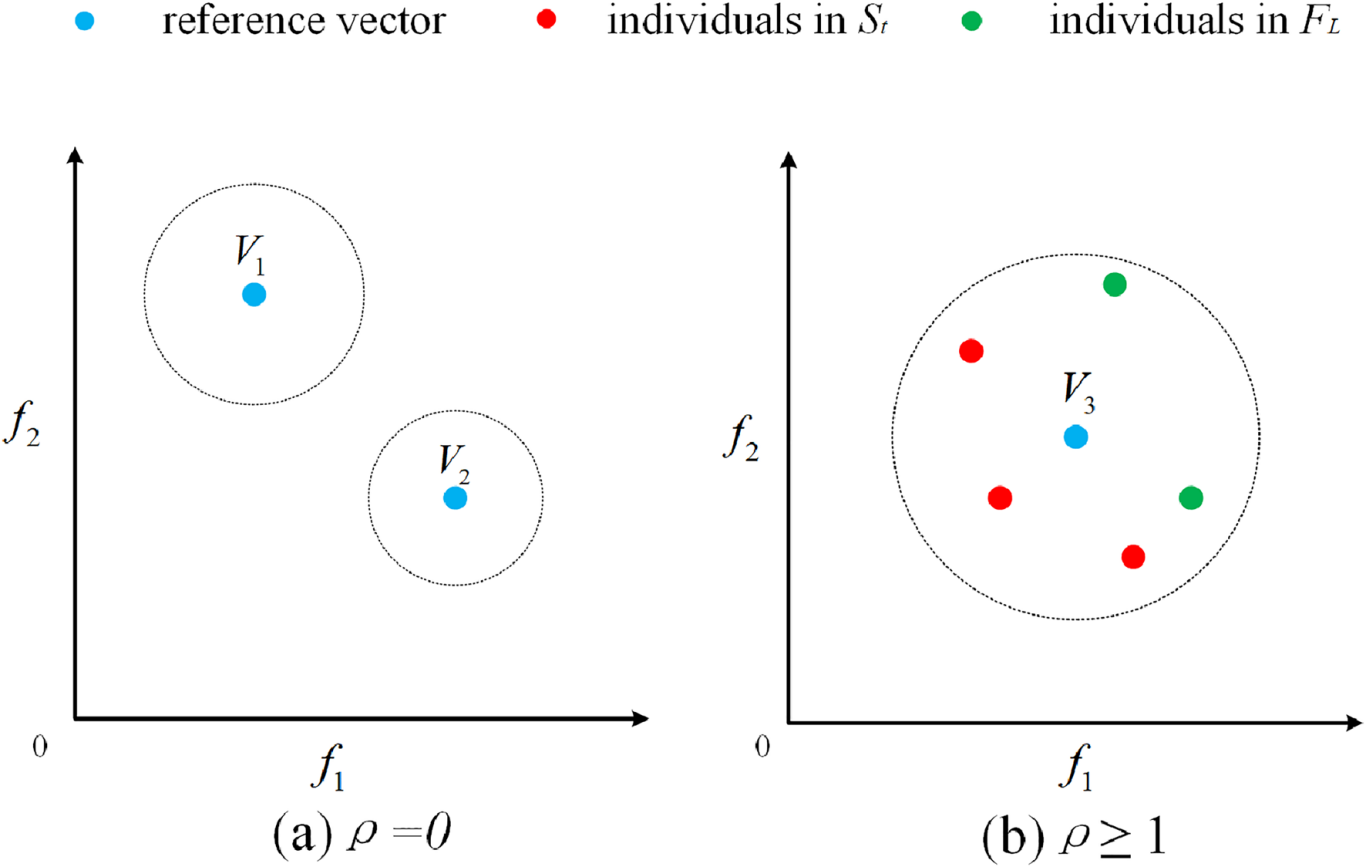
Description of selecting individuals for each reference vector.

The selection strategy is based on a set of predefined reference vectors and the vertical distance between the individuals and the reference vectors. Objective values and reference points are initially normalized to align them on the same hyperplane^45^. Each individual is associated with a reference vector based on the minimum vertical distance. Next, select individuals for each reference vector. Referring to Fig. 1a, if the number of individuals (*ρ*) in *S*_*t*_ corresponding to the reference vector is 0 and no individuals in *F*_*L*_ belong to this reference vector (such as *V*_1_ and *V*_2_), this reference vector is no longer considered. But in cases where *ρ* ≥ 1(as shown in Fig. 1b), randomly select an individual from individuals of *F*_*L*_ belonging to this reference vector. This iterative process continues until the number of individuals in *S*_*t*_ reaches *N*.

### Angle-penalized distance

The primary difficulty of high-dimensional EMOPs is achieving convergence to PF while simultaneously preserving solution diversity. Several studies have been proposed to address such questions, including the aforementioned RVEA algorithm. RVEA introduces an angle-penalized distance as a selection criterion, which can effectively balance diversity and convergence according to the experimental results in^17^.

APD relies on a set of reference vectors that partition the objective space into multiple subspaces, the selection of each subspace is carried out independently. First, all individuals will be converted to objective vectors according to the following formula.

2 $$ f^{\prime}_{i} = f_{i} - Z_{\min } $$

where *f*_*i*_ represents the objective value of the *i*^th^ individual and *Z*_min_ is the set of the minimum objective value of *f*. The translation of the objective function is to ensure that the initial point of the reference vector is always the origin, and all the translated objective values fall within the positive orthant. After the translation, APD adopts the acute angle between individuals and the reference vectors, as well as the length of the objective vectors to assess the overall performance of solutions by using the following formula:

3 $$ d^{i} = \left( {1 + P\left( {\theta^{i} } \right)} \right) \cdot \left\| {\overline{v}^{i} } \right\| $$

where *P*(*θ*^*i*^) and ||^*i*^|| measure the diversity performance and convergence performance, respectively. *v*^*i*^ represents the objective vector corresponding to the *i*^th^ individual. *θ*^*i*^ represents the angle between the individual and the reference vector.

4 $$ P\left( {\theta^{i} } \right) = M \cdot \left( {\frac{t}{{t_{\max } }}} \right)^{\alpha } \cdot \frac{{\theta^{i} }}{{\gamma_{v} }} $$

where *M* represents the number of objectives, *t* represents the current generation, and *t*_max_ represents the maximum generation. The angle *γ*^*v*^ is used to normalize the angle, which is important when the distribution of reference vectors is too dense or too sparse. The value of *α* controls the changing rate of *P*(*θ*^*i*^) and a larger *α* means that more emphasis will be allocated to the convergence performance compared to the diversity performance.

### Radial basis function

Radial Basis Function (RBF)^8,46,47^ is a discrete multivariate data interpolation model. The value of the function depends on the Euclidean distance from the sample point to the measured point. RBF model is constructed by linearly stacking the basis function values. The basic expression of the basis function is as follows:

5 $$ \hat{y}\left( x \right) = \sum\limits_{i = 1}^{n} {\lambda_{i} \Phi } \left( {\left\| {x - x_{i} } \right\|} \right) $$

where *λ*_*i*_ is the weight coefficient of the *i*^th^ basis function; ||*x*-*x*_*i*_|| is the Euclidean distance between the measured vector point and the sample point *x*_*i*_; *n* is the number of variables, and *Φ*(·) is the radial basis function.

The critical steps of using the RBF model are involve selecting the basis function and calculating the weight coefficient. The choice of basis functions and coefficients directly impacts the fitting accuracy and local characteristics of the surrogate model^48^. In this paper, an RBF model with multi-quadratic functions is adopted to assist the search work.

According to various research, each surrogate model has its unique characteristics and applications. For example, Kriging and SVM are suitable for low-dimensional problems, but when the dimension of the problem is increasing, the training process becomes computationally expensive^29^. Comparatively speaking, RBF is less sensitive to dimensionality and offers fast modeling speed, which is advantageous for high-dimensional EMOPs.

## Proposed algorithm

In this section, the details of the proposed TSDEA will be given. The overall framework of TSDEA is presented first, followed by its two-stage selection strategy. Finally, the archive updating strategy is introduced.

### Algorithm framework

Algorithm 1 outlines the primary framework of the proposed algorithm TSDEA. The algorithm composes of five components: initialization, establishment and updating of the surrogate model, generation of candidate offspring, reference vector based two-stage selection strategy, and finally an archive updating strategy. Reference vector based two-stage selection strategy is comprised of Algorithm 2 and Algorithm 3, which select individuals according to their dominant relationship and APD, respectively. Algorithm 4 is primarily applied for archive management, which is ultimately employed to update the surrogate model.

1. *Initialization* During the initialization phase, an initial population of size *N* is generated using Latin hypercube sampling (LHS) and evaluated using the original fitness function. The initial population will be included in the archive *D*_*s*_ and *D* respectively, where *D*_*s*_ is for subsequent surrogate model updates, and *D* is the final returned solution set. In addition, a set of uniformly distributed reference vectors is generated.
2. *Building and updating the RBF Model* In the first iteration, the individuals in *D*_*s*_ are used to construct the RBF model for the evaluation of offspring individuals. In each subsequent iteration, new individuals that exhibit a balance between diversity and convergence are selected and added to the *D*_*s*_ for retraining models.
3. *Candidate offspring generation* In each generation, *N* offspring solutions are generated using the simulated binary crossover (SBX)^49^ and the polynomial mutation^50^. The selection of parents is based on a tournament strategy that considers dominant relationships and crowding distances, ensuring a higher chance of selecting optimal individuals.
4. *Reference vector based two-stage selection strategy* At this stage, we apply two sub-selection strategies, the dominance-based and the APD-based selection strategy. For the first stage, an improved dominance-based NSGAIII based on predefined generations is applied to search the decision and objective spaces exhaustively. The second stage involves selecting individuals based on either their APD value or crowding distance.
5. *Deleting redundant individuals in the archive D*_*s*_ After the selected solutions are evaluated, they are added to *D*_*s*_ and *D*. To prevent an increase in model retraining time due to an excessive number of individuals, the total number of individuals in *D*_*s*_ is limited. To control the number, we introduce the extreme point. Whenever the number of individuals in *D*_*s*_ exceeds the limit, all individuals are re-selected by the uncertainty calculated by the Euclidean distance from the extreme point.

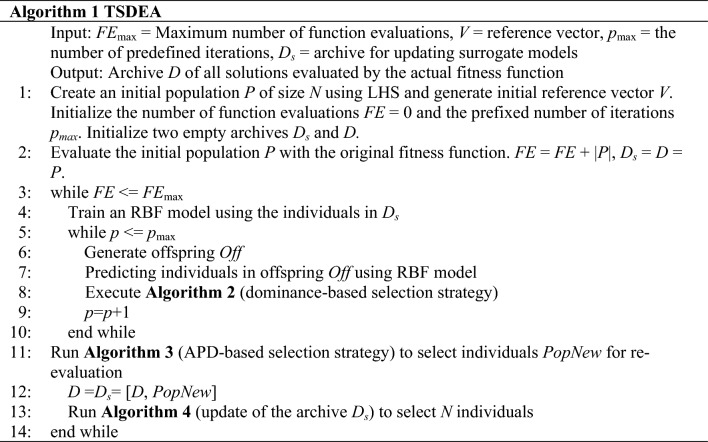


### Reference vector based two-stage selection strategy

As shown in Algorithm 1, individuals need to be selected from population *P* and its offspring for re-evaluation after offspring generation. In the algorithm, a reference vector based two-stage selection strategy is adopted. The following is a detailed introduction to the two-stage algorithm.

#### Dominance-based selection strategy

For high-dimensional EMOPs with extensive decision space, we need to explore the decision space thoroughly and find the optimal solutions that can balance diversity and convergence. In this stage, this paper utilizes an improved NSGAIII to search the entire space. As described in^9^, NSGAIII demonstrates excellent convergence and diversity maintenance in the population iteration process, and maintain uniform distribution of solutions on the non-dominant layer to avoid the algorithm falling into the local optimum. Besides, we have made appropriate enhancements to NSGAIII to help the selected individuals distribute more evenly.

**Figure 2:**
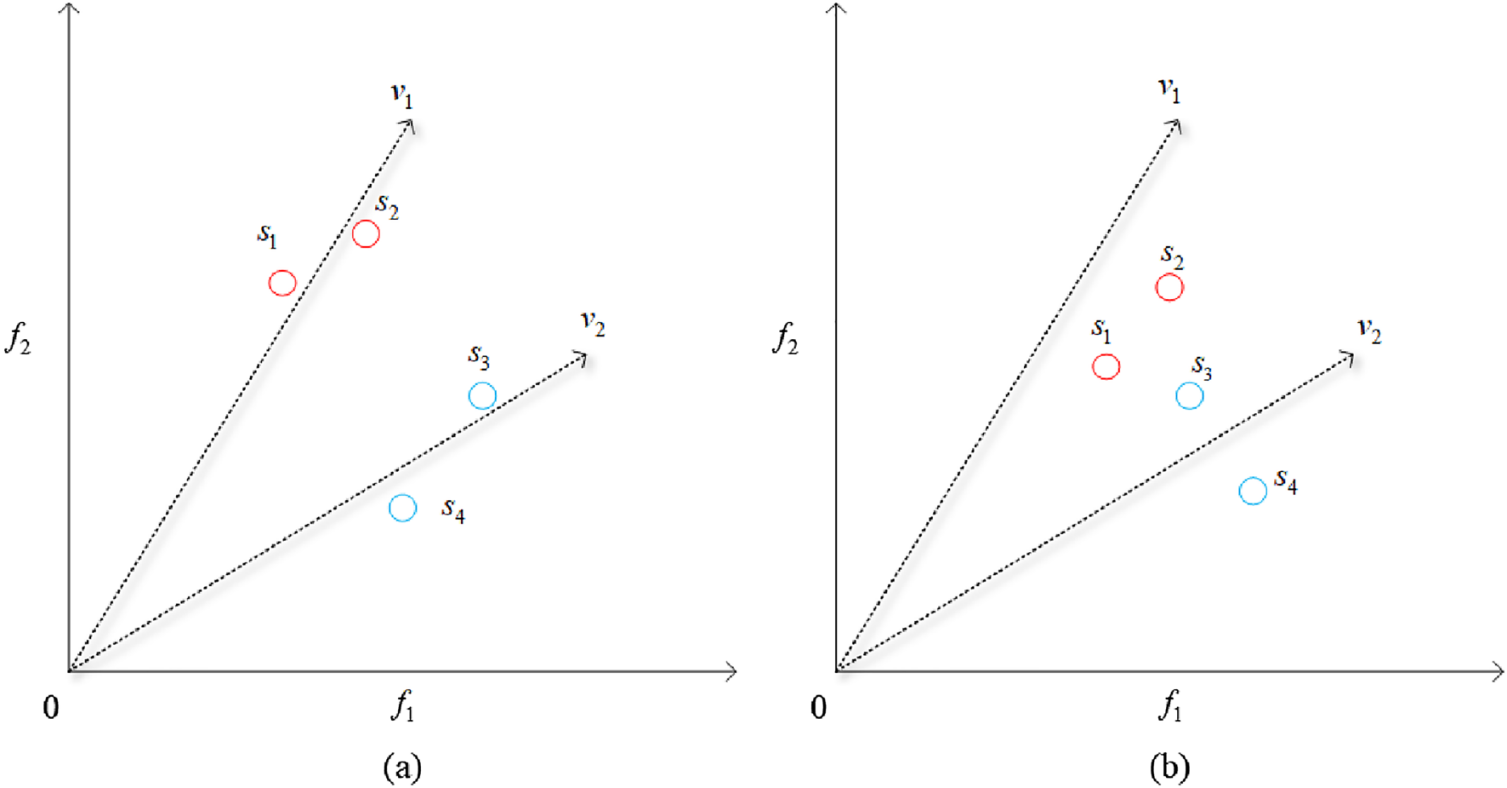
Different cases while selecting individuals. (**a**) Shows that the angle between the individual and the reference vector is small. (**b**) Shows that the angle between the individual and the reference vector is uneven.

As explained in “Preliminary”, NSGAIII adopts a niche operation to select individuals, but when multiple individuals are associated with the same reference vector, the results become random. Based on this, we propose a new method that takes the angles between individuals and the reference vector as the selection criterion. Smaller angles indicate the proximity of individuals to the reference vector in space, resulting in a more uniformly distributed solution set. For instance, consider the two situations depicted in Fig. 2.

In Fig. 2a, when the angle between an individual and its corresponding reference vector is small, a uniform individual distribution can be obtained by selecting any individual. However, when some individuals are distant from the reference vector, random selection may make the selected individuals gradually concentrate. As shown in Fig. 2b, if *s*_*2*_ and *s*_*3*_ are selected, the gap between the selected individuals is minimal, and the diversity of the population cannot be improved. If individuals are selected according to the angle, such as *s*_*1*_ and *s*_*4*_, a relatively more balanced distribution among individuals can be achieved. Therefore, selecting individuals according to the angle is more conducive to maintain population distribution.

Although the improved NSGAIII has better performance, it cannot fully explore the whole space in the limited FEs. Therefore, prefixed generation is incorporated into the algorithm, allowing the evolutionary algorithm to search enough on the fitness landscape until neither convergence nor diversity can be further enhanced. In the dominance-based selection strategy proposed in this paper, the number of prefixed generations is determined by sensitivity analysis of parameters.

The pseudocode of the algorithm is shown in Algorithm 2. The population *P* and *Off* evaluated by the surrogate model are merged into a new population *P*_*t*_ of size 2*N*. In this step, we need to select *N* individuals as the parent for the next iteration. First, individuals are adaptively normalized and assigned (lines 2–5). Subsequently, individuals are selected from the subspace associated with each reference vector to proceed to the next iteration (lines 7–9). The selection operation is no longer random, but the angles between the individuals and the reference vector. The individuals with smaller angles are preferentially selected.
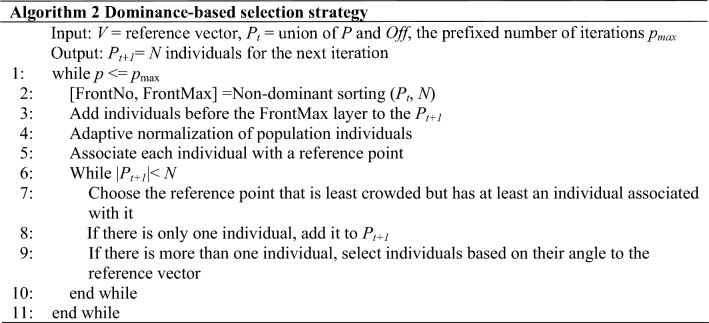


#### APD-based selection strategy

After the first stage of selection based on the approximation of the surrogate model, we need to further screen the individuals. The selected individuals are evaluated using the original fitness function and used to enhance the accuracy of the surrogate model, which is important for optimizing the overall performance of the algorithm.

At this stage, we utilize two criteria of APD and crowding distance to select individuals. Through massive simulation experiments in RVEA, APD can well adapt to the changes in the number of objectives and dimensions, thereby ensuring an optimal balance between convergence and diversity. However, it is hard to guarantee that all reference vectors have associated individuals in the optimization process. To tackle this problem, we take the crowding distance as another selection criterion. The specific application of the two criteria is based on the number of empty reference vectors *V*_*e*_. When *V*_*e*_ surpasses a certain threshold ***ɛ***, it indicates a concentrated distribution of individuals. Continuing to select individuals from these densely distributed areas would result in the population gradually converging towards a small region^23^. In this case, we employ the crowding distance to select individuals. Crowding distance is an indicator describing the degree of crowding between an individual and its neighboring points. A larger crowding distance signifies a more dispersed distribution of individuals within the population. Selecting individuals with larger crowding distances can help us search for regions with higher uncertainty and better maintain the diversity of the population.

As demonstrated in Algorithm 3, the crowding distance (line 1) is first calculated. The objective function is then translated to guarantee that the initial point of the reference vector is always the origin and that the objective values for all translations are in the positive quadrant (line 2). Next, individuals are assigned and APD values are calculated according to the APD calculation method described in “Preliminary” (lines 3–4). Finally, the number of empty reference points is counted. If *V*_*e*_ doesn’t exceed the threshold ***ɛ***, the individual with the smaller APD value is selected, otherwise, the individual with the larger crowding distance is selected (lines 7–11).
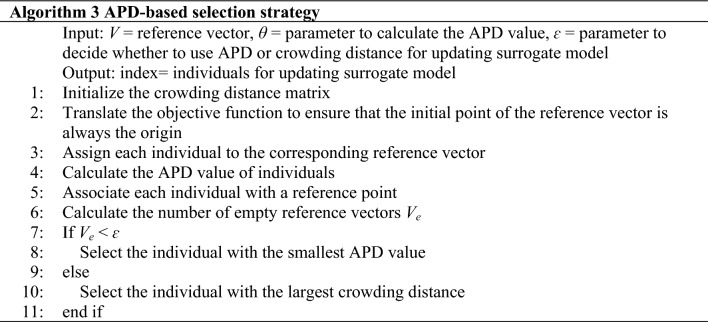


### Archive updating strategy

The solutions selected in the reference vector based two-stage selection strategy will be evaluated by the actual fitness function and used for the model update. However, the growing number of samples during iterations introduces another challenge known as archive management. It is widely acknowledged that the cost of training the surrogate model is positively correlated with the number of samples. Though we have chosen the RBF model which is relatively unaffected by the number of samples, additional samples may still cause unnecessary computation costs and accuracy reduction.

To avoid this problem, we implement an archive updating strategy to eliminate inferior solutions from the archive. As mentioned above, we always keep two archives (*D* and *D*_*s*_) in the process of optimization. The size of *D*_*s*_ for updating the surrogate model is limited. If the total number of newly added individuals in *D*_*s*_ surpasses *N*, then redundant individuals need to be deleted.

In the archive updating strategy, the retained individuals need to satisfy two requirements: (1) fulfill convergence and diversity; (2) fulfill the accuracy of the RBF model. To achieve this, we employ the dominant relationship and the Euclidean distance between individuals and the extreme point as the criteria. Individuals with better fitness values are selected according to the dominance relation, which can accelerate convergence to PF, and the Euclidean distance was introduced as a measure of uncertainty.

6 $$ \begin{array}{*{20}c} {z_{i}^{extreme} = \left( {f_{1} \left( x \right), \ldots ,f_{i} \left( x \right), \ldots ,f_{k} \left( x \right)} \right),} \\ {x = \arg \min (f_{i} ),} \\ {f_{i} \in PF,1 \le i \le k} \\ \end{array} $$

Extreme points consist of minimum values for all objectives. There are *k* extreme points for a MOP with *k* objectives. The definition of the *i*th extreme point is given as shown in Eq. (6). The individuals which are distant from these extreme points exhibit higher uncertainty to improve the diversity and the accuracy of the models. Consequently, we choose individuals with the maximum Euclidean distance from the extreme point. Next, we will introduce the specific usage of the two criteria.

First, all the individuals are non-dominant. Individuals before the last layer are added to the next generation (line 3). For the individuals in the last layer, calculate the Euclidean distance between the individuals and the extreme points (lines 4–6). A larger distance indicates a higher likelihood of an individual being distant from the Pareto Front (PF), thereby avoiding the trap of local optima during the optimization process.
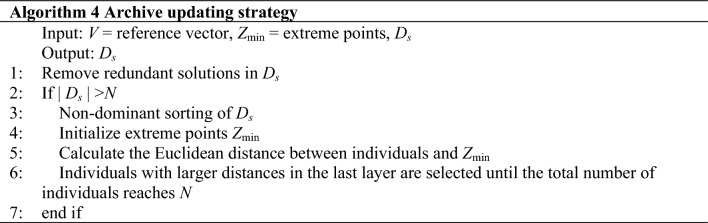


## Numerical experiments

In this section, we examine the performance of the algorithm by comparing TSDEA with five state-of-the-art algorithms, namely AB-MOEA, EDN-ARMOEA, CSEA, K-RVEA, and CPS-MOEA.

This experiment is performed based on objective number M = {2, 3} and decision variables *D* = {20, 50, 100}. The Wilcoxon rank sum test is utilized to compare the results obtained at a significance level of 0.05 for TSDEA and the other compared algorithms in 30 independent runs. The symbols "+" and "−" indicate that the performance of this algorithm is significantly better or significantly lower than that of the comparison algorithm, while "≈" indicates that there is no statistically significant difference between the comparison algorithms.

For comparisons, the inverted generational distance (IGD)^51^ and the modified IGD (IGD+^52^) metrics are adopted for evaluating the performance of the compared algorithms. Both IGD and IGD+ are comprehensive measures of diversity and convergence. More details about both indicators can be found in Supplementary Information A.

### Experimental settings

1. *Problem settings *In this experiment, we mainly employ three types of test problems, namely DTLZ (DTLZ1-7), WFG (WFG1-9), and ZDT (ZDT1-4, ZDT6)^53^. For the 3-objective problem, we choose the DTLZ and WFG problems. Since the WFG problem can only be applied to the problem of 3 objectives and above, we choose the ZDT benchmark problems in the 2-objective problem.
2. *Comparison algorithms* Five comparison algorithms are selected for the comparison test, among which the approximation-based SAEAs include AB-MOEA, K-RVEA, and EDN-ARMOEA, and the classification-based SAEAs include CSEA and CPS-MOEA. All algorithms used for comparison in this experiment are from the PlatEMO platform.
  *AB-MOEA* The parameter controlling the rate of change of penalty *α* = 2, number of generations before updating Kriging models *w*_max_ = 20, Number of re-evaluated solutions at each generation *u* = 5;
  *K-RVEA* the penalty control parameter *α* = 2, the frequency of reference vector adaptation *fr* = 0.1, *u* = 5, *δ* = 0.05N, and *w*_max_ = 20 for parameters to manage Kriging models;
  *EDN-ARMOEA* a diversity threshold parameter *δ* = 0.08, the maximum number of generations for AR-MOEA iteration = 20, the number of FEs at each generation *k* = 5; for ANN parameters, *J* = *K* = 40, *wd* = 10^–5^;
  *CSEA* the number of hidden neurons of FNN *H* = 10, the number of reference solutions *K* = 6, and the number of solutions evaluated by surrogate model gmax = 3000;
  *CPS-MOEA* the number of generated offspring for each solution *M* = 3;
3. Experiment parameter
  The initial population size and data size *N* = 100,
  The maximum number of expensive function evaluations *FEs* = 500.
  The number of independent runs *Run* = 30.
  The number of generations before updating the Kriging models *p*_max_ = 20.
  The parameter determining which criteria *ɛ* = 0.1.
  The parameter of the RBF model: The type of basis function is multiquadric. The range of input variables is [− 1, 1].

### Comparison results and analysis

To assess the efficiency of the proposed algorithm in handling high-dimensional EMOPs, TSDEA is compared with AB-MOEA, EDN-ARMOEA, CSEA, K-RVEA, and CPS-MOEA with varying numbers of dimensions and objectives. In addition to the DTLZ benchmarks, we set ZDT for the 2 objectives problem and WFG for the 3 objectives problem separately. To adequately present the dominance and quality of the obtained solutions from all compared SAEAs, we take IGD and IGD+ as the performance indicator in the following experiments. To further illustrate the advantage of the proposed algorithm, the non-dominant solution set obtained by the six algorithms on DTLZ2, DTLZ5, and ZDT1 are visualized. The results clearly demonstrate the outstanding performance of our algorithm.

We compare the proposed algorithm with other algorithms in terms of both 2 and 3 objectives. The statistical results of IGD+ values are shown in Tables 1 and 2, and IGD results are in Supplementary Tables 1 and 2. As indicated in the tables, TSDEA outperformed the other algorithms in 20 (in Table 1) and 29 (in Table 2) test problems respectively, accounting for more than half of all test problems. These results show the competitiveness of TSDEA over its compared algorithms. However, the IGD+ values of TSDEA for DTLZ1, DTLA3, and DTLZ6 with different decision variables are not the best, which is similar to the 3-objective problems. This observation can be attributed to the fact that DTLZ1 and DTLZ3 have multi-modal fitness landscapes with multiple locally optimal solutions, while DTLZ6 has a large number of separated Pareto optimal regions in the decision space. In conclusion, the proposed algorithm shows the best overall performance.

**Table 1:** Statistical results for IGD+ values obtained by AB-MOEA, EDN-ARMOEA, CSEA, K-RVEA, CPS-MOEA, and TSDEA for 2 objectives with the same number of real FEs.

Problem	D	ABSAEA	EDNARMOEA	CSEA	KRVEA	CPSMOEA	mmNSGA3
DTLZ1	20	3.2556e + 2 (3.16e + 1) +	4.1311e + 2 (4.42e + 1) -	**2.3490e + 2 (4.69e + 1) + **	2.9026e + 2 (4.49e + 1) +	3.2327e + 2 (3.58e + 1) +	3.5576e + 2 (3.51e + 1)
	50	1.4293e + 3 (5.55e + 1) -	1.4263e + 3 (5.19e + 1) -	1.4121e + 3 (6.05e + 1) -	1.4112e + 3 (5.74e + 1) -	**1.0967e + 3 (6.79e + 1) + **	1.1479e + 3 (1.00e + 2)
	100	3.0909e + 3 (1.11e + 2) -	3.0893e + 3 (1.15e + 2) -	3.0570e + 3 (1.25e + 2) -	3.0789e + 3 (8.78e + 1) −	**2.3942e + 3 (1.33e + 2) + **	2.5567e + 3 (1.99e + 2)
DTLZ2	20	1.4612e−1 (5.80e−2) −	4.5062e−1 (7.67e−2) −	2.8933e−1 (5.38e−2) −	9.4837e−2 (2.63e−2) −	6.4658e−1 (1.20e−1) −	**8.7954e**−**3 (2.61e**−**3)**
	50	2.6976e + 0 (1.35e−1) −	2.6756e + 0 (1.27e−1) −	2.6630e + 0 (1.77e−1) −	2.7239e + 0 (1.31e−1) −	1.7422e + 0 (2.22e−1) −	**1.0892e + 0 (7.23e**−**1)**
	100	6.0187e + 0 (2.49e−1) =	6.0700e + 0 (1.22e−1) =	6.0328e + 0 (2.30e−1) =	6.0484e + 0 (2.22e−1) =	**3.7983e + 0 (5.13e**−**1) + **	5.8511e + 0 (7.16e−1)
DTLZ3	20	8.2035e + 2 (1.10e + 2) =	1.1228e + 3 (9.07e + 1) −	**5.4002e + 2 (1.24e + 2) + **	7.8450e + 2 (1.04e + 2) =	8.3661e + 2 (9.61e + 1) =	8.2279e + 2 (1.50e + 2)
	50	3.7907e + 3 (1.79e + 2) −	3.7994e + 3 (1.63e + 2) −	3.7880e + 3 (1.63e + 2) −	3.7906e + 3 (1.83e + 2) −	**2.7593e + 3 (2.11e + 2) + **	3.3714e + 3 (2.83e + 2)
	100	8.4823e + 3 (1.93e + 2) −	8.4506e + 3 (2.26e + 2) −	8.5084e + 3 (1.59e + 2) −	8.3981e + 3 (2.59e + 2) −	**6.1885e + 3 (4.14e + 2) + **	7.6854e + 3 (4.49e + 2)
DTLZ4	20	5.3740e−1 (2.10e−1) −	4.0549e−1 (1.27e−1) −	4.0927e−1 (9.33e−2) −	5.8782e−1 (1.93e−1) −	7.5736e−1 (1.03e−1) −	**2.8425e**−**1 (1.07e**−**1)**
	50	2.8799e + 0 (1.54e−1) −	2.9489e + 0 (1.45e−1) −	2.9350e + 0 (1.35e−1) −	2.8787e + 0 (1.89e−1) −	1.8766e + 0 (2.80e−1) −	**6.1794e**−**1 (2.45e**−**1)**
	100	6.3738e + 0 (1.95e−1) −	6.2669e + 0 (2.83e−1) −	6.3464e + 0 (2.15e−1) −	6.2800e + 0 (2.17e−1) −	3.8968e + 0 (6.19e−1) =	**3.3585e + 0 (1.72e + 0)**
DTLZ5	20	1.1681e−1 (5.97e−2) −	4.8097e−1 (7.71e−2) −	2.8101e−1 (5.56e−2) −	1.0125e−1 (3.14e−2) −	5.9786e−1 (8.79e−2) −	**8.3429e**−**3 (2.15e**−**3)**
	50	2.6643e + 0 (1.80e−1) −	2.6799e + 0 (1.61e−1) −	2.7236e + 0 (1.02e−1) −	2.6832e + 0 (1.26e−1) −	1.6733e + 0 (2.34e−1) −	**1.2395e + 0 (7.48e**−**1)**
	100	6.1251e + 0 (1.71e−1) =	6.0268e + 0 (1.64e−1) =	6.1207e + 0 (2.12e−1) =	6.0698e + 0 (1.87e−1) =	**3.7871e + 0 (4.80e**−**1) + **	1.2395e + 0 (7.48e−1)
DTLZ6	20	1.0592e + 1 (7.66e−1) =	1.2772e + 1 (6.61e−1) −	1.0740e + 1 (1.00e + 0) =	**9.9699e + 0 (7.83e**−**1) = **	1.0690e + 1 (7.29e−1) =	1.0408e + 1 (1.27e + 0)
	50	4.2713e + 1 (2.21e−1) −	4.2707e + 1 (2.09e−1) −	4.2732e + 1 (1.95e−1) −	4.2775e + 1 (1.73e−1) −	**3.2139e + 1 (1.03e + 0) + **	3.5754e + 1 (1.86e + 0)
	100	8.7329e + 1 (2.72e−1) −	8.7281e + 1 (2.73e−1) −	8.7252e + 1 (2.87e−1) −	8.7292e + 1 (2.55e−1) −	**6.6993e + 1 (2.38e + 0) + **	8.1536e + 1 (8.15e−1)
DTLZ7	20	1.8117e−1 (1.90e−1) =	9.0368e−1 (3.12e−1) −	1.4103e + 0 (7.67e−1) −	**2.4884e**−**2 (3.84e**−**3) + **	4.8609e + 0 (6.11e−1) −	1.5783e−1 (1.16e−1)
	50	6.2801e + 0 (2.89e−1) −	6.2177e + 0 (2.19e−1) −	6.1532e + 0 (2.81e−1) −	6.2691e + 0 (3.34e−1) −	6.1380e + 0 (4.03e−1) −	**9.5338e**−**1 (2.88e**−**1)**
	100	6.6340e + 0 (2.09e−1) −	6.7401e + 0 (1.77e−1) −	6.6160e + 0 (2.47e−1) −	6.6182e + 0 (2.02e−1) −	6.6379e + 0 (2.95e−1) −	**2.7511e + 0 (3.52e**−**1)**
ZDT1	20	1.3233e−1 (7.32e−2) −	3.2990e−1 (4.05e−2) −	3.4718e−1 (1.43e−1) −	2.2203e−2 (2.18e−3) −	1.6923e + 0 (2.31e−1) −	**2.1106e**−**2 (1.26e**−**2)**
	50	2.2840e + 0 (9.95e−2) −	2.2254e + 0 (1.22e−1) −	2.2678e + 0 (1.05e−1) −	2.2005e + 0 (1.27e−1) −	2.1098e + 0 (1.15e−1) −	**2.9505e**−**1 (6.60e**−**2)**
	100	2.3996e + 0 (6.06e−2) −	2.4149e + 0 (8.40e−2) −	2.4036e + 0 (8.23e−2) −	2.3994e + 0 (6.75e−2) −	2.3219e + 0 (1.06e−1) −	**1.0704e + 0 (1.25e−1)**
ZDT2	20	1.1392e−1 (1.17e−1) =	7.0967e−1 (6.73e−2) −	1.1157e + 0 (2.18e−1) −	**2.0551e**−**2 (3.28e**−**3) = **	2.8177e + 0 (3.16e−1) −	8.3851e−2 (1.07e−1)
	50	3.5999e + 0 (1.47e−1) −	3.5897e + 0 (1.73e−1) −	3.5737e + 0 (1.24e−1) −	3.6370e + 0 (1.41e−1) −	3.5095e + 0 (2.46e−1) −	**3.0681e**−**1 (1.60e**−**1)**
	100	3.9112e + 0 (8.95e−2) −	3.9217e + 0 (7.56e−2) −	3.9287e + 0 (8.40e−2) −	3.9227e + 0 (9.42e−2) −	3.9018e + 0 (1.07e−1) −	**1.3235e + 0 (3.47e**−**1)**
ZDT3	20	1.3686e−1 (1.03e−1) +	2.7898e−1 (5.24e−2) =	3.0405e−1 (8.06e−2) =	**2.0999e**−**2 (4.16e**−**3) + **	1.4611e + 0 (1.60e−1) −	3.2249e−1 (9.47e−2)
	50	1.7971e + 0 (1.26e−1) −	1.8360e + 0 (1.42e−1) −	1.8521e + 0 (1.39e−1) −	1.8522e + 0 (1.19e−1) −	1.8155e + 0 (1.80e−1) −	**7.5171e**−**1 (1.54e**−**1)**
	100	1.9810e + 0 (9.04e−2) −	1.9166e + 0 (7.71e−2) −	1.9578e + 0 (8.87e−2) −	1.9386e + 0 (7.04e−2) −	1.9874e + 0 (1.16e−1) −	**1.1763e + 0 (1.07e**−**1)**
ZDT4	20	1.0415e + 2 (1.81e + 1) −	1.5577e + 2 (1.61e + 1) −	1.2052e + 2 (2.00e + 1) −	1.1969e + 2 (1.85e + 1) −	1.9716e + 2 (1.69e + 1) −	**5.6809e + 1 (3.29e + 1)**
	50	6.5618e + 2 (2.22e + 1) −	6.5620e + 2 (2.80e + 1) −	6.5980e + 2 (2.81e + 1) −	6.6189e + 2 (3.28e + 1) −	6.1394e + 2 (3.67e + 1) −	**3.1509e + 2 (6.96e + 1)**
	100	1.4540e + 3 (4.25e + 1) −	1.4599e + 3 (3.16e + 1) −	1.4551e + 3 (2.92e + 1) −	1.4545e + 3 (3.24e + 1) −	1.2864e + 3 (5.70e + 1) −	**1.0662e + 3 (8.10e + 1)**
ZDT6	20	**1.3647e + 0 (3.93e**−**1) + **	5.0961e + 0 (2.22e−1) −	5.4291e + 0 (3.75e−1) −	1.6456e + 0 (7.47e−1) +	6.7600e + 0 (1.80e−1) −	2.2488e + 0 (3.08e−1)
	50	7.3745e + 0 (6.81e−2) −	7.3886e + 0 (6.98e−2) −	7.3877e + 0 (7.37e−2) −	7.3770e + 0 (8.79e−2) −	7.2416e + 0 (1.06e−1) −	**4.5043e + 0 (3.92e**−**1)**
	100	7.5377e + 0 (3.70e−2) −	7.5230e + 0 (6.05e−2) −	7.5309e + 0 (3.40e−2) −	7.5486e + 0 (3.33e−2) −	7.4651e + 0 (8.38e−2) −	**6.0294e + 0 (1.56e**−**1)**
**+/**−**/=**		3/27/6	0/33/3	2/30/4	4/27/5	9/24/3	

The best results are highlighted in bold.

**Table 2:** Statistical results for IGD + values obtained by AB-MOEA, EDN-ARMOEA, CSEA, K-RVEA, CPS-MOEA, and TSDEA for 3 objectives with the same number of real FEs.

Problem	D	ABSAEA	EDNARMOEA	CSEA	KRVEA	CPSMOEA	TSDEA
DTLZ1	20	2.8377e + 2 (4.18e + 1) −	3.1773e + 2 (3.29e + 1) −	**1.9572e + 2 (3.09e + 1) + **	2.9757e + 2 (4.07e + 1) −	2.5304e + 2 (3.40e + 1) =	2.5177e + 2 (3.90e + 1)
	50	1.2029e + 3 (5.78e + 1) −	1.1786e + 3 (6.02e + 1) −	1.2007e + 3 (6.85e + 1) −	1.2028e + 3 (5.17e + 1) −	**9.2159e + 2 (6.21e + 1) + **	1.0601e + 3 (7.56e + 1)
	100	2.5830e + 3 (1.02e + 2) =	2.6066e + 3 (8.88e + 1) =	2.5773e + 3 (9.33e + 1) =	2.5892e + 3 (9.36e + 1) =	**2.0772e + 3 (1.32e + 2) + **	2.6183e + 3 (1.52e + 2)
DTLZ2	20	3.7368e−1 (6.11e−2) −	7.8888e−1 (5.34e−2) −	3.5342e−1 (7.05e−2) −	6.7551e−1 (8.89e−2) −	7.2468e−1 (9.03e−2) −	**1.0799e**−**1 (2.29e**−**2)**
	50	2.7232e + 0 (1.26e−1) −	2.7178e + 0 (1.49e−1) −	2.7187e + 0 (1.06e−1) −	2.7262e + 0 (1.00e−1) −	**1.8272e + 0 (2.90e**−**1) + **	2.2003e + 0 (3.02e−1)
	100	6.1104e + 0 (1.75e−1) −	6.0763e + 0 (2.14e−1) −	6.0986e + 0 (1.69e−1) −	6.0221e + 0 (2.40e−1) −	**3.7748e + 0 (6.32e**−**1) + **	5.5076e + 0 (3.72e−1)
DTLZ3	20	8.1850e + 2 (9.30e + 1) =	1.0593e + 3 (9.83e + 1) −	**5.4428e + 2 (9.12e + 1) + **	8.0779e + 2 (1.27e + 2) =	7.5407e + 2 (8.48e + 1) =	7.8757e + 2 (1.21e + 2)
	50	3.7581e + 3 (1.54e + 2) −	3.7402e + 3 (1.49e + 2) −	3.7792e + 3 (1.60e + 2) −	3.6825e + 3 (1.81e + 2) −	**2.7113e + 3 (2.10e + 2) + **	3.2691e + 3 (2.82e + 2)
	100	8.3160e + 3 (2.44e + 2) −	8.3178e + 3 (2.26e + 2) −	8.3788e + 3 (2.56e + 2) −	8.4012e + 3 (1.70e + 2) −	**6.0293e + 3 (5.49e + 2) + **	7.5396e + 3 (4.07e + 2)
DTLZ4	20	5.9489e−1 (1.56e−1) −	5.4263e−1 (1.07e−1) −	**3.5993e**−**1 (6.91e**−**2) = **	8.0141e−1 (1.54e−1) −	8.7691e−1 (1.07e−1) −	3.9862e−1 (8.90e−2)
	50	3.0091e + 0 (1.33e−1) −	3.0107e + 0 (1.46e−1) −	2.9674e + 0 (1.65e−1) −	2.9792e + 0 (1.44e−1) −	2.0761e + 0 (2.28e−1) −	**1.1918e + 0 (4.75e**−**1)**
	100	6.2932e + 0 (2.51e−1) −	6.3591e + 0 (2.13e−1) −	6.2433e + 0 (3.10e−1) −	6.3384e + 0 (2.00e−1) −	**4.1180e + 0 (4.97e**−**1) + **	4.7921e + 0 (7.66e−1)
DTLZ5	20	2.8049e−1 (5.70e−2) −	6.5502e−1 (8.01e−2) −	3.5172e−1 (8.06e−2) −	5.0959e−1 (1.12e−1) −	6.1211e−1 (7.01e−2) −	**3.6663e**−**2 (8.70e**−**3)**
	50	2.6478e + 0 (1.26e−1) −	2.6128e + 0 (1.65e−1) −	2.6151e + 0 (1.75e−1) −	2.6487e + 0 (1.46e−1) −	**1.8445e + 0 (2.87e**−**1) + **	2.0239e + 0 (3.62e−1)
	100	6.0182e + 0 (1.66e−1) −	6.0197e + 0 (2.16e−1) −	6.0197e + 0 (2.08e−1) −	6.0220e + 0 (2.01e−1) −	**3.8176e + 0 (4.58e**−**1) + **	5.6228e + 0 (3.05e−1)
DTLZ6	20	1.0193e + 1 (6.66e−1) =	1.4021e + 1 (5.08e−1) −	1.3653e + 1 (9.14e−1) −	**8.7982e + 0 (6.45e**−**1) + **	1.0100e + 1 (8.54e−1) =	1.0106e + 1 (8.07e−1)
	50	4.1888e + 1 (2.54e−1) −	4.1857e + 1 (2.92e−1) −	4.1934e + 1 (2.04e−1) −	4.1936e + 1 (2.07e−1) −	**3.0712e + 1 (1.61e + 0) + **	3.9430e + 1 (1.05e + 0)
	100	8.6484e + 1 (2.20e−1) −	8.6423e + 1 (3.17e−1) −	8.6414e + 1 (3.16e−1) −	8.6402e + 1 (2.84e−1) −	**6.7582e + 1 (2.25e + 0) + **	8.3949e + 1 (9.41e−1)
DTLZ7	20	2.9149e−1 (2.57e−1) +	1.8748e + 0 (5.79e−1) −	2.6080e + 0 (8.35e−1) −	**8.0765e**−**2 (7.12e**−**3) + **	6.8441e + 0 (6.46e−1) −	3.9655e−1 (1.16e−1)
	50	9.3695e + 0 (4.80e−1) −	9.3282e + 0 (5.33e−1) −	9.3144e + 0 (4.46e−1) −	9.2600e + 0 (5.87e−1) −	8.8788e + 0 (6.28e−1) −	**1.6948e + 0 (4.08e**−**1)**
	100	9.9101e + 0 (3.66e−1) −	9.9868e + 0 (3.17e−1) −	9.8956e + 0 (3.93e−1) −	9.9129e + 0 (3.82e−1) −	9.9689e + 0 (3.44e−1) −	**4.0322e + 0 (6.86e**−**1)**
WFG1	20	1.7682e + 0 (1.41e−1) =	1.8841e + 0 (6.68e−2) −	**1.5608e + 0 (4.97e**−**2) + **	1.6873e + 0 (1.06e−1) =	2.1749e + 0 (6.89e−2) −	1.7178e + 0 (6.60e−2)
	50	2.1798e + 0 (3.92e−2) −	2.1800e + 0 (3.99e−2) −	2.1668e + 0 (3.74e−2) −	2.1866e + 0 (3.62e−2) −	2.1726e + 0 (4.08e−2) −	**1.7517e + 0 (8.83e**−**2)**
	100	2.1399e + 0 (4.32e−2) −	2.1398e + 0 (3.14e−2) −	2.1380e + 0 (3.82e−2) −	2.1297e + 0 (6.43e−2) −	2.1890e + 0 (4.28e−2) −	**1.7368e + 0 (7.76e**−**2)**
WFG2	20	**4.6832e**−**1 (7.01e**−**2) + **	6.7783e−1 (3.65e−2) −	4.7347e−1 (4.88e−2) +	5.2556e−1 (8.44e−2) +	7.3697e−1 (3.62e−2) −	6.2701e−1 (6.09e−2)
	50	8.2622e−1 (2.26e−2) −	8.4181e−1 (3.07e−2) −	8.2902e−1 (3.17e−2) −	8.3482e−1 (3.28e−2) −	8.1111e−1 (3.23e−2) −	**7.1664e**−**1 (3.00e**−**2)**
	100	7.9937e−1 (1.24e−2) −	8.0253e−1 (1.21e−2) −	8.0586e−1 (1.01e−2) −	8.0315e−1 (1.17e−2) −	8.2161e−1 (3.24e−2) −	**7.7195e**−**1 (2.38e**−**2)**
WFG3	20	**5.3483e**−**1 (5.74e**−**2) = **	7.1054e−1 (2.06e−2) −	5.9672e−1 (3.96e−2) =	6.8531e−1 (2.83e−2) −	7.1392e−1 (3.54e−2) −	5.7025e−1 (5.98e−2)
	50	7.7479e−1 (1.27e−2) =	7.7204e−1 (1.03e−2) =	7.7281e−1 (1.15e−2) =	7.7620e−1 (1.05e−2) =	8.2662e−1 (3.64e−2) −	**7.7108e**−**1 (2.07e**−**2)**
	100	8.0149e−1 (5.94e−3) +	8.0014e−1 (6.80e−3) +	**7.9855e**−**1 (5.02e**−**3) + **	7.9998e−1 (6.88e−3) +	8.5612e−1 (3.47e−2) −	8.3122e−1 (1.22e−2)
WFG4	20	3.7885e−1 (1.41e−2) −	4.7765e−1 (8.91e−3) −	3.8485e−1 (2.47e−2) −	4.6788e−1 (1.67e−2) −	4.7811e−1 (1.62e−2) −	**3.5559e**−**1 (3.83e**−**2)**
	50	5.9425e−1 (3.70e−2) −	5.9784e−1 (2.28e−2) −	5.8958e−1 (2.61e−2) −	5.9419e−1 (2.59e−2) −	5.4109e−1 (1.23e−2) −	**4.6740e**−**1 (2.37e**−**2)**
	100	5.6382e−1 (2.21e−2) −	5.6800e−1 (2.09e−2) −	5.6628e−1 (2.48e−2) −	5.6533e−1 (2.22e−2) −	5.6517e−1 (1.26e−2) −	**5.1529e**−**1 (1.90e**−**2)**
WFG5	20	5.0294e−1 (4.89e−2) −	6.0558e−1 (2.00e−2) −	4.1618e−1 (3.18e−2) −	**3.5924e**−**1 (3.29e**−**2) + **	5.3751e−1 (2.26e−2) −	3.8537e−1 (4.36e−2)
	50	7.3912e−1 (7.68e−3) −	7.4212e−1 (8.50e−3) −	7.4348e−1 (7.34e−3) −	7.4152e−1 (7.88e−3) −	6.1829e−1 (2.08e−2) −	**5.8332e**−**1 (3.79e**−**2)**
	100	7.4456e−1 (4.59e−3) −	7.4316e−1 (3.81e−3) −	7.4498e−1 (4.71e−3) −	7.4388e−1 (5.05e−3) −	**6.5238e**−**1 (1.58e**−**2) + **	6.8311e−1 (2.63e−2)
WFG6	20	7.4754e−1 (3.87e−2) −	8.1620e−1 (1.74e−2) −	5.9865e−1 (4.18e−2) =	6.7313e−1 (4.14e−2) −	8.5811e−1 (2.78e−2) −	**5.8559e**−**1 (6.24e**−**2)**
	50	9.1586e−1 (1.18e−2) −	9.1389e−1 (1.03e−2) −	9.1750e−1 (1.20e−2) −	9.1677e−1 (1.06e−2) −	9.6917e−1 (1.68e−2) −	**8.0509e**−**1 (5.25e**−**2)**
	100	9.2375e−1 (5.81e−3) −−	9.2435e−1 (5.91e−3) −	9.2394e−1 (6.97e−3) −	9.2452e−1 (5.96e−3) −	9.9976e−1 (1.85e−2) −	**8.9024e**−**1 (2.72e**−**2)**
WFG7	20	4.9000e−1 (2.68e−2) =	6.2300e−1 (1.16e−2) −	5.1177e−1 (3.32e−2) −	6.3967e−1 (1.24e−2) −	6.3042e−1 (1.77e−2) −	**4.6640e**−**1 (4.72e**−**2)**
	50	6.8819e−1 (8.02e−3) −	6.9093e−1 (1.30e−2) −	6.9258e−1 (8.65e−3) −	6.9117e−1 (8.11e−3) −	7.0440e−1 (1.71e−2) −	**6.1482e**−**1 (3.28e**−**2)**
	100	6.8773e−1 (4.28e−3) −	6.8943e−1 (5.35e−3) −	6.8872e−1 (5.83e−3) −	6.8955e−1 (4.95e−3) −	7.4123e−1 (1.79e−2) −	**6.8255e**−**1 (1.08e**−**2)**
WFG8	20	**5.4647e**−**1 (3.79e**−**2) + **	6.9437e−1 (1.80e−2) −	5.9470e−1 (6.37e−2) =	6.6032e−1 (1.96e−2) −	7.5424e−1 (2.52e−2) −	5.8752e−1 (4.63e−2)
	50	7.8780e−1 (1.69e−2) −	7.8458e−1 (1.71e−2) −	7.9211e−1 (1.63e−2) −	7.9064e−1 (1.31e−2) −	7.9562e−1 (2.21e−2) −	**6.7104e**−**1 (2.73e**−**2)**
	100	7.5193e−1 (9.07e−3) −	7.5356e−1 (8.34e−3) −	7.5323e−1 (1.02e−2) −	7.4997e−1 (8.69e−3) −	8.0820e−1 (1.48e−2) −	**7.0777e**−**1 (2.16e**−**2)**
WFG9	20	6.5404e−1 (6.78e−2) −	8.0876e−1 (4.35e−2) −	6.6440e−1 (8.02e−2) −	7.5853e−1 (5.95e−2) −	7.3172e−1 (4.92e−2) −	**5.6977e**−**1 (1.10e**−**1)**
	50	9.4366e−1 (2.57e−2) −	9.3203e−1 (2.20e−2) −	9.4855e−1 (2.58e−2) −	9.4803e−1 (2.51e−2) −	8.5468e−1 (5.66e−2) −	**8.0587e**−**1 (7.22e**−**2)**
	100	9.5590e−1 (9.97e−3) −	9.5152e−1 (1.06e−2) −	9.5291e−1 (9.51e−3) −	9.5801e−1 (1.45e−2) −	9.3799e−1 (5.04e−2) −	**8.9854e**−**1 (5.57e**−**2)**
+ /−/ =		4/37/7	1/45/2	5/37/6	5/39/4	12/33/3	

The best results are highlighted in bold.

**Figure 3:**
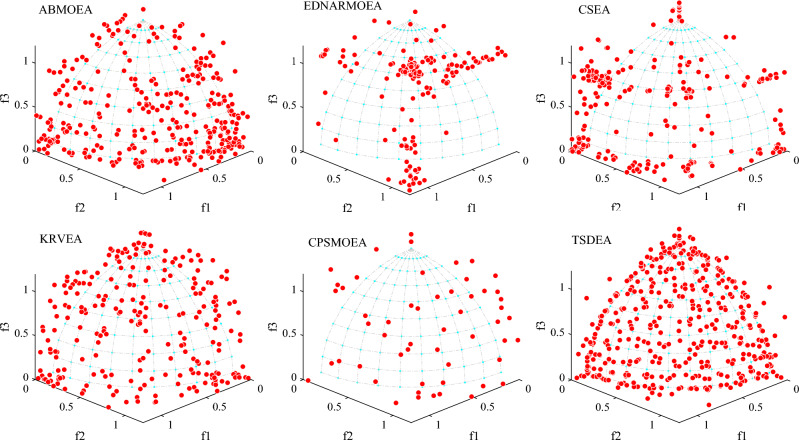
The non-dominant solutions obtained by each algorithm on 3-objective DTLZ2 in the run associated with the median IGD value.

**Figure 4:**
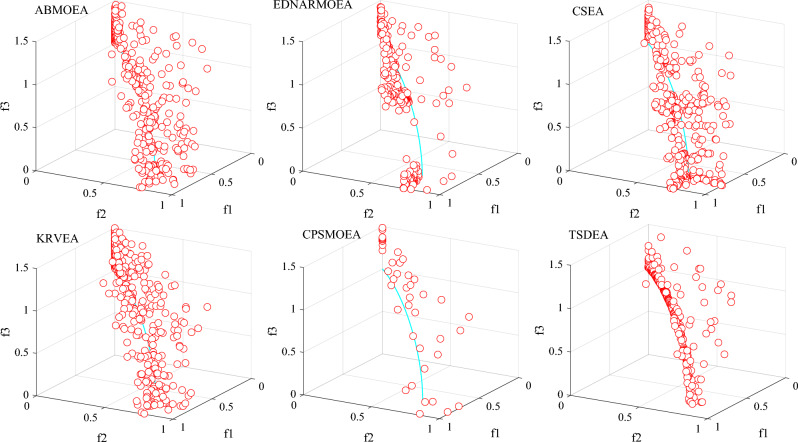
The non-dominant solutions obtained by each algorithm on 3-objective DTLZ5 in the run associated with the median IGD value.

**Figure 5:**
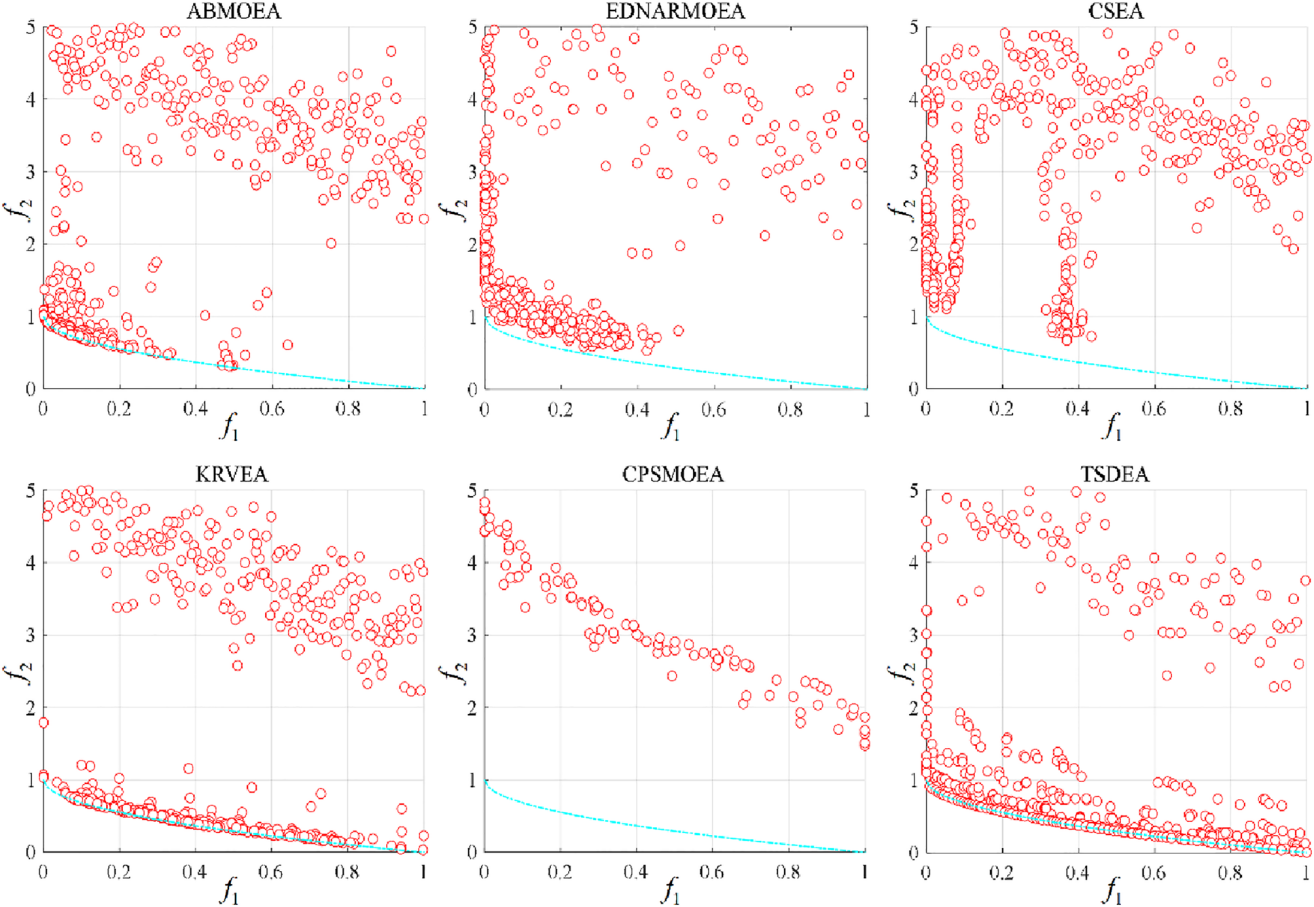
The non-dominant solutions obtained by each algorithm on 2-objective ZDT1 in the run associated with the median IGD value.

To further compare the advantages of TSDEA, we visualize the non-dominant solution sets acquired by the six algorithms on 3-objective DTLZ2 and DTLZ5 in Figs. 3 and 4. Additionally, Fig. 5 illustrates the distribution of non-dominant solutions of the six algorithms for the 2-objective ZDT1 problem. Upon reviewing Figs. 3 and 4, non-dominant solutions obtained from K-RVEA and AB-MOEA are much closer to the true PF and exhibit a more balanced distribution the other three comparison algorithms, but they are weaker than TSDEA. Figure 5 reveals that none of the non-dominant solution sets can cover the true PF perfectly except for TSDEA. This reaffirms that TSDEA algorithm can obtain a set of non-dominant solutions with the best convergence and the most uniform distribution in the above problems. Therefore, the performance of TSDEA is better than the other five comparison algorithms.

### Runtime comparison

For the currently widely used surrogate model, the training time is closely related to the number of objectives and decision variables. Moreover, the training sample will also influences the computing time of the surrogate model. For the TSDEA proposed in this paper, we utilize the RBF model which is less insensitive to dimension and sample sizes.

**Figure 6:**
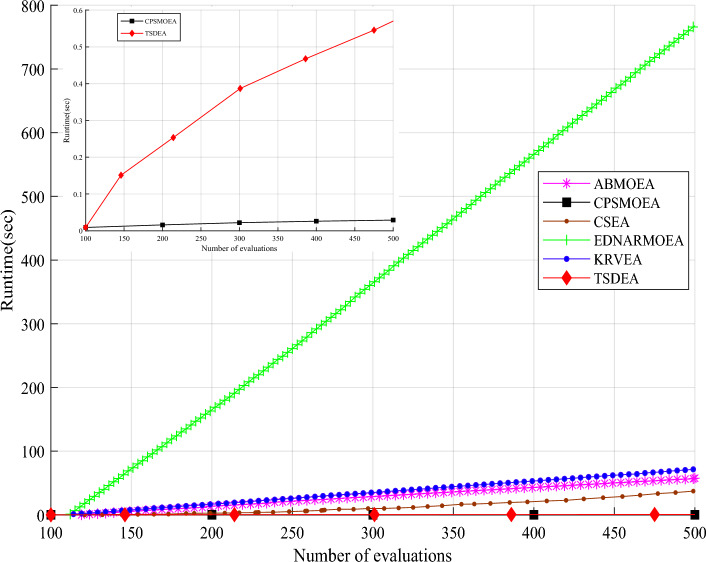
Runtime over the number of evaluations in AB-MOEA, CPS-MOEA, CSEA, EDN-ARMOEA, K-RVEA, and TSDEA.

To assess the computational efficiency of TSDEA, we compare the running time of six algorithms AB-MOEA, CPS-MOEA, CSEA, EDN-ARMOEA, K-RVEA, and TSDEA for DTLZ2 problem with 3 objectives, and the results are shown in Fig. 6.

Observing Fig. 6, it becomes apparent that the running time of the six algorithms increases linearly with the increase of the number of evaluations, where the runtime of EDN-ARMOEA increases more rapidly and AB-MOEA, CSEA, and K-RVEA have a slower growth rate. Conversely, CPS-MOEA and TSDEA show the slowest growth. From the details, the speed of CPS-MOEA is lower than that of TSDEA. The main reason is that CPS-MOEA uses KNN to predict the candidate solutions, and the computational complexity of KNN is much lower than that of the RBF method. In a word, the computational efficiency of TSDEA exceeds that of most algorithms.

### Sensitivity analysis of parameters

TSDEA mainly contains two parameters, namely *ɛ* and *p*_max_. In Algorithm 3, *ɛ* is utilized to determine whether to update the surrogate model with APD or crowding distance. *p*_max_ specifies how often the surrogate should be updated, that is, when the surrogate should be updated. We analyze the sensitivity of parameters in TSDEA through the experiment of DTLZ5.

**Figure 7:**
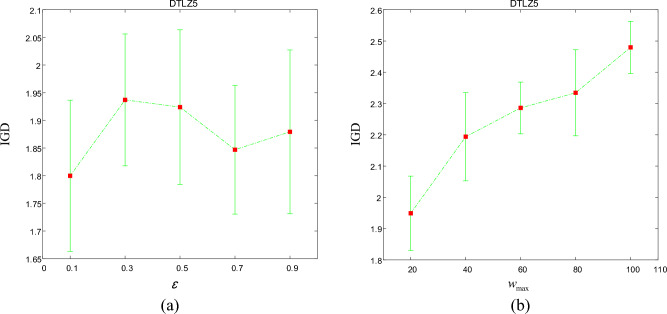
Average IGD profile plots over the different parameter values in TSDEA on the 3-objective DTLZ5 based on 30 independent runs. a Is the performance profile plots of parameter ɛ. b Is the performance profile plots of parameter *p*_*max*_.

When *p*_max_ = 20, We set *ɛ* to 0.1,0.3,0.5,0.7,0.9 for comparison. Figure 7a summarizes the average IGD values with different values of *ɛ*. As you can see from the figure, the IGD value is minimal when *ɛ* = 0.1, so set *ɛ* to 0.1 as a general setting for all test instances.

Next, we will test the effect of *p*_max_ by setting different parameters. In this experiment, we set *p*_max_ to 20, 40, 60, 80, and 100. The experimental results are shown in Fig. 7b. When *p*_max_ is 20, the average IGD value obtained is the smallest, so 20 is taken as the final value of *p*_max_.

### Effects of the individual selection criteria

In the APD based selection strategy, we employ two individual selection criteria. To assess the efficiency of these criteria, we compare TSDEA with two variants: TSDEA (APD) and TSDEA (CD). TSDEA (APD) solely utilizes the APD criterion for individual selection, while TSDEA (CD) solely employs crowding distance for individual selection. The experiment is conducted on WFG problems and the statistical results are shown in Supplementary Table 3. The results demonstrate that TSDEA outperformed the two variants on all benchmark problems. In WFG3 and WFG6, TSDEA is significantly better than the two variants and TSDEA also achieved comparable or superior results in other test questions, which indicates that the combination of the two selection criteria is superior to any single criterion. Therefore, the validity of the two individual selection criteria cannot be ignored.

### Effects of the two-stage selection strategies

To further investigate the effectiveness of the two selection strategies proposed in this paper, TSDEA is compared with the two variants, as shown below.

- TSDEA(D): only use the dominance-based selection strategy
- TSDEA(A): only use APD based selection strategy

The experiment is conducted on DTLZ1-7 with 3 objectives and 20 dimensions, and the statistical results can be found in Supplementary Table 1. As can be seen from the table, except for DTLZ4, TSDEA obtained the best results on the other six test problems. On the problem of DTLZ4, TSDEA (A) performs slightly better than TSDEA. The reason may be that the point density on the real PF of DTLZ4 has a large deviation. To obtain better results, the algorithm must maintain the proper distribution of candidate solutions. However, the dominance-based selection strategy primarily prioritizes solution convergence, thus not delivering a noticeable improvement for DTLZ4. Nevertheless, when considering overall performance, the combination of both selection strategies outperforms any single-strategy algorithm.

## Conclusions

In this paper, a two-stage dominance-based surrogate-assisted evolution algorithm for solving high-dimensional expensive multi-objective optimization (TSDEA) is proposed. The RBF model is applied in the algorithm to approximate the objective function to reduce the actual evaluation cost. To enhance diversity and convergence, we employ a reference vector based two-stage search method in the algorithm. The dominance-based selection strategy and the APD-based selection strategy are used to select individuals with better diversity and convergence, respectively. These individuals are re-evaluated through the actual fitness function and utilized to update the surrogate model. When updating the surrogate model, considering the cost of model retraining, we keep the number of individuals for updating limited.

We evaluate the proposed algorithm on benchmarks with 20, 50, and 100 decision variables. Comprehensive results show that TSDEA outperforms five state-of-the-art algorithms (AB-MOEA, EDN-ARMOEA, CSEA, K-RVEA, and CPS-MOEA) with the same parameter settings and fixed evaluations.

Although the proposed algorithm is competitive on most test problems, this work is preliminary and we will further improve the predictive accuracy of the surrogate model with more than 3 objectives and 100 dimensions in future work. Moreover, the application of the proposed algorithm to some real-world high-dimensional EMOPs will be considered in our future work.

### Supplementary Information

Supplementary Information.

## Data Availability

The datasets used and/or analyzed during the current study are available from the corresponding author on reasonable request.
