# Supplementary material for: A two-stage dominance-based surrogate-assisted evolution algorithm for high-dimensional expensive multi-objective optimization
Source: Sci Rep. 2023 Aug 13;13:13163. doi: 10.1038/s41598-023-40019-6 (PMC10423721; doi:10.1038/s41598-023-40019-6)
Supplement: Supplementary file 1 — Supplementary Information. [file 41598_2023_40019_MOESM1_ESM.docx]

**Supplementary Information**

**A Performance indicators**

IGD

For comparisons, the inverted generational distance (IGD) [52] is adopted for evaluating the performance of the compared algorithms. IGD is defined as follows:

$$IGD\left(A,Z\right)=\frac{1}{Z}\left(\sum_{j=1}^{\left|Z\right|} d_{j}\left(z,a\right)^{p}\right)^{1/p}$$

Among them, *Z* is the point set uniformly distributed on the real Pareto surface, and |*Z*| is the number of individuals in the point set. *A* is the optimal Pareto optimal solution set obtained by the algorithm. And *d*(*z, a*) is the minimum Euclidean distance from individual *z* to population *A* in *Z*. IGD evaluates the comprehensive performance of the algorithm by calculating the average value of the minimum distance between the real Pareto surface point set and the acquired population. The smaller the value, the better the overall performance of the algorithm including convergence and distribution performance.

IGD+

The modified inverted generational distance (IGD+) is a variant of IGD. IGD+ does not meet the Pareto criteria and shows some distinct advantages over the original IGD. The modified inverted generational distance (IGD+) indicator can be viewed as follows:

$$IGD^{+}\left(A,Z\right)=\frac{1}{Z}\left(\sum_{j=1}^{\left|Z\right|} d_{j}^{+}\left(z,a\right)^{p}\right)^{1/p}$$

where a ∈ A , z ∈ Z, A is the Pareto set approximation and Z is the reference set. *d^+^*(*z*, *a*) is defined as

$$d_{j}^{+}\left(z,a\right)=\sqrt{\sum_{i=1}^{m} \left(\max\left(a_{i}-z_{i},0\right)\right)^{2}}$$

Therefore, a low IGD+ value means that the set A has a better approximation to the real PF if we consider the reference set as PF.

Statistical results for IGD values obtained by AB-MOEA, EDN-ARMOEA, CSEA, K-RVEA, CPS-MOEA, and TSDEA for 2 objectives with the same number of real FEs. The best results are highlighted in blue.

**B Experimental Results**

**Supplementary Table 1.** Statistical results for IGD values obtained by AB-MOEA, EDN-ARMOEA, CSEA, K-RVEA, CPS-MOEA, and TSDEA for 2 objectives with the same number of real FEs. The best results are highlighted in blue.

| Problem | D | AB-MOEA | EDN-ARMOEA | CSEA | K-RVEA | CPS-MOEA | TSDEA |
| --- | --- | --- | --- | --- | --- | --- | --- |
| DTLZ1 | 20 | 3.2556e+2 (3.16e+1) + | 4.1311e+2 (4.42e+1) - | 2.3490e+2 (4.69e+1) + | 2.9026e+2 (4.49e+1) + | 3.2327e+2 (3.58e+1) + | 3.5576e+2 (3.51e+1) |
|  | 50 | 1.4293e+3 (5.55e+1) - | 1.4263e+3 (5.19e+1) - | 1.4121e+3 (6.05e+1) - | 1.4112e+3 (5.74e+1) - | 1.0967e+3 (6.79e+1) + | 1.1479e+3 (1.00e+2) |
|  | 100 | 3.0909e+3 (1.11e+2) - | 3.0893e+3 (1.15e+2) - | 3.0570e+3 (1.25e+2) - | 3.0789e+3 (8.78e+1) - | 2.3942e+3 (1.33e+2) + | 2.5567e+3 (1.99e+2) |
| DTLZ2 | 20 | 1.5773e-1 (5.80e-2) - | 4.6880e-1 (7.32e-2) - | 3.1802e-1 (5.06e-2) - | 1.0346e-1 (2.75e-2) - | 6.5038e-1 (1.20e-1) - | 1.3338e-2 (4.25e-3) |
|  | 50 | 2.6986e+0 (1.34e-1) - | 2.6769e+0 (1.26e-1) - | 2.6633e+0 (1.77e-1) - | 2.7243e+0 (1.31e-1) - | 1.7433e+0 (2.23e-1) - | 1.0927e+0 (7.23e-1) |
|  | 100 | 6.0190e+0 (2.49e-1) = | 6.0702e+0 (1.22e-1) = | 6.0341e+0 (2.28e-1) = | 6.0485e+0 (2.22e-1) = | 3.7983e+0 (5.13e-1) + | 5.8517e+0 (7.16e-1) |
| DTLZ3 | 20 | 8.2035e+2 (1.10e+2) = | 1.1228e+3 (9.07e+1) - | 5.4002e+2 (1.24e+2) + | 7.8450e+2 (1.04e+2) = | 8.3661e+2 (9.61e+1) = | 8.2279e+2 (1.50e+2) |
|  | 50 | 3.7907e+3 (1.79e+2) - | 3.7994e+3 (1.63e+2) - | 3.7880e+3 (1.63e+2) - | 3.7906e+3 (1.83e+2) - | 2.7593e+3 (2.11e+2) + | 3.3714e+3 (2.83e+2) |
|  | 100 | 8.4823e+3 (1.93e+2) - | 8.4506e+3 (2.26e+2) - | 8.5084e+3 (1.59e+2) - | 8.3981e+3 (2.59e+2) - | 6.1885e+3 (4.14e+2) + | 7.6854e+3 (4.49e+2) |
| DTLZ4 | 20 | 5.5235e-1 (2.16e-1) = | 5.1661e-1 (1.86e-1) = | 4.8900e-1 (1.21e-1) = | 6.2932e-1 (1.95e-1) = | 8.7897e-1 (1.03e-1) - | 5.0941e-1 (2.56e-1) |
|  | 50 | 2.9345e+0 (1.47e-1) - | 3.0084e+0 (1.39e-1) - | 2.9883e+0 (1.40e-1) - | 2.9362e+0 (1.81e-1) - | 1.9895e+0 (2.64e-1) - | 8.1163e-1 (1.45e-1) |
|  | 100 | 6.4054e+0 (1.92e-1) - | 6.3019e+0 (2.80e-1) - | 6.3785e+0 (2.11e-1) - | 6.3063e+0 (2.14e-1) - | 3.9584e+0 (6.08e-1) = | 3.4431e+0 (1.62e+0) |
| DTLZ5 | 20 | 1.3106e-1 (5.86e-2) - | 4.9595e-1 (7.44e-2) - | 3.0502e-1 (5.26e-2) - | 1.1305e-1 (3.38e-2) - | 6.0271e-1 (8.78e-2) - | 1.3244e-2 (3.94e-3) |
|  | 50 | 2.6648e+0 (1.80e-1) - | 2.6806e+0 (1.61e-1) - | 2.7245e+0 (1.02e-1) - | 2.6843e+0 (1.26e-1) - | 1.6751e+0 (2.34e-1) - | 1.2440e+0 (7.47e-1) |
|  | 100 | 6.1256e+0 (1.72e-1) = | 6.0269e+0 (1.64e-1) = | 6.1211e+0 (2.12e-1) = | 6.0698e+0 (1.87e-1) = | 3.7871e+0 (4.80e-1) + | 6.0934e+0 (4.28e-1) |
| DTLZ6 | 20 | 1.0592e+1 (7.65e-1) = | 1.2774e+1 (6.61e-1) - | 1.0743e+1 (1.00e+0) = | 9.9729e+0 (7.82e-1) = | 1.0691e+1 (7.29e-1) = | 1.0413e+1 (1.26e+0) |
|  | 50 | 4.2713e+1 (2.21e-1) - | 4.2707e+1 (2.09e-1) - | 4.2732e+1 (1.95e-1) - | 4.2775e+1 (1.73e-1) - | 3.2140e+1 (1.03e+0) + | 3.5754e+1 (1.86e+0) |
|  | 100 | 8.7329e+1 (2.72e-1) - | 8.7281e+1 (2.73e-1) - | 8.7252e+1 (2.87e-1) - | 8.7292e+1 (2.55e-1) - | 6.6993e+1 (2.38e+0) + | 8.1536e+1 (8.15e-1) |
| DTLZ7 | 20 | 2.1858e-1 (2.26e-1) = | 9.2033e-1 (3.30e-1) - | 1.4322e+0 (7.83e-1) - | 2.9089e-2 (3.76e-3) + | 4.8612e+0 (6.11e-1) - | 1.7079e-1 (1.31e-1) |
|  | 50 | 6.2801e+0 (2.89e-1) - | 6.2178e+0 (2.19e-1) - | 6.1536e+0 (2.81e-1) - | 6.2691e+0 (3.34e-1) - | 6.1380e+0 (4.03e-1) - | 9.5882e-1 (2.82e-1) |
|  | 100 | 6.6340e+0 (2.09e-1) - | 6.7401e+0 (1.77e-1) - | 6.6160e+0 (2.47e-1) - | 6.6182e+0 (2.02e-1) - | 6.6379e+0 (2.95e-1) - | 2.7523e+0 (3.53e-1) |
| ZDT1 | 20 | 2.0405e-1 (1.19e-1) - | 3.3796e-1 (4.68e-2) - | 3.6436e-1 (1.65e-1) - | 2.3540e-2 (2.43e-3) - | 1.6925e+0 (2.31e-1) - | 2.2579e-2 (1.40e-2) |
|  | 50 | 2.2841e+0 (9.95e-2) - | 2.2256e+0 (1.22e-1) - | 2.2680e+0 (1.05e-1) - | 2.2008e+0 (1.27e-1) - | 2.1098e+0 (1.15e-1) - | 2.9610e-1 (6.85e-2) |
|  | 100 | 2.3996e+0 (6.06e-2) - | 2.4149e+0 (8.40e-2) - | 2.4036e+0 (8.22e-2) - | 2.3994e+0 (6.75e-2) - | 2.3219e+0 (1.06e-1) - | 1.0704e+0 (1.25e-1) |
| ZDT2 | 20 | 1.5340e-1 (1.74e-1) = | 8.1898e-1 (1.02e-1) - | 1.2364e+0 (2.00e-1) - | 2.8896e-2 (5.08e-3) = | 2.8215e+0 (3.17e-1) - | 1.2316e-1 (1.86e-1) |
|  | 50 | 3.6078e+0 (1.43e-1) - | 3.5918e+0 (1.73e-1) - | 3.5826e+0 (1.26e-1) - | 3.6419e+0 (1.44e-1) - | 3.5121e+0 (2.45e-1) - | 4.8722e-1 (2.64e-1) |
|  | 100 | 2.3996e+0 (6.06e-2) - | 2.4149e+0 (8.40e-2) - | 2.4036e+0 (8.22e-2) - | 2.3994e+0 (6.75e-2) - | 2.3219e+0 (1.06e-1) - | 1.0704e+0 (1.25e-1) |
| ZDT3 | 20 | 1.5823e-1 (1.18e-1) + | 2.8311e-1 (5.46e-2) = | 3.0795e-1 (8.19e-2) = | 3.0680e-2 (4.95e-3) + | 1.4612e+0 (1.60e-1) - | 3.2489e-1 (9.54e-2) |
|  | 50 | 1.7971e+0 (1.26e-1) - | 1.8360e+0 (1.42e-1) - | 1.8521e+0 (1.39e-1) - | 1.8523e+0 (1.19e-1) - | 1.8155e+0 (1.80e-1) - | 7.5178e-1 (1.54e-1) |
|  | 100 | 3.9148e+0 (8.86e-2) - | 3.9276e+0 (7.30e-2) - | 3.9315e+0 (8.53e-2) - | 3.9258e+0 (9.55e-2) - | 3.9028e+0 (1.07e-1) - | 1.3872e+0 (3.65e-1) |
| ZDT4 | 20 | 1.0415e+2 (1.81e+1) - | 1.5577e+2 (1.61e+1) - | 1.2052e+2 (2.00e+1) - | 1.1969e+2 (1.85e+1) - | 1.9716e+2 (1.69e+1) - | 5.6812e+1 (3.29e+1) |
|  | 50 | 6.5618e+2 (2.22e+1) - | 6.5620e+2 (2.80e+1) - | 6.5980e+2 (2.81e+1) - | 6.6189e+2 (3.28e+1) - | 6.1394e+2 (3.67e+1) - | 3.1509e+2 (6.96e+1) |
|  | 100 | 1.4540e+3 (4.25e+1) - | 1.4599e+3 (3.16e+1) - | 1.4551e+3 (2.92e+1) - | 1.4545e+3 (3.24e+1) - | 1.2864e+3 (5.70e+1) - | 1.0662e+3 (8.10e+1) |
| ZDT6 | 20 | 1.3834e+0 (3.98e-1) + | 5.1020e+0 (2.20e-1) - | 5.4365e+0 (3.75e-1) - | 1.6732e+0 (7.39e-1) + | 6.7603e+0 (1.80e-1) - | 2.2489e+0 (3.08e-1) |
|  | 50 | 7.3748e+0 (6.79e-2) - | 7.3887e+0 (6.97e-2) - | 7.3879e+0 (7.39e-2) - | 7.3770e+0 (8.79e-2) - | 7.2416e+0 (1.06e-1) - | 4.5061e+0 (3.91e-1) |
|  | 100 | 7.5377e+0 (3.70e-2) - | 7.5231e+0 (6.05e-2) - | 7.5309e+0 (3.41e-2) - | 7.5487e+0 (3.33e-2) - | 7.4651e+0 (8.37e-2) - | 7.4651e+0 (8.37e-2) - |
| +/-/= | | 3/26/7 | 0/32/4 | 2/29/5 | 4/26/6 | 9/24/3 |  |

**Supplementary Table 2.** Statistical results for IGD values obtained by AB-MOEA, EDN-ARMOEA, CSEA, K-RVEA, CPS-MOEA, and TSDEA for 3 objectives with the same number of real FEs. The best results are highlighted in blue.

| Problem | D | AB-MOEA | EDN-ARMOEA | CSEA | K-RVEA | CPS-MOEA | TSDEA |
| --- | --- | --- | --- | --- | --- | --- | --- |
| DTLZ1 | 20 | 2.8377e+2 (4.18e+1) - | 3.1773e+2 (3.29e+1) - | 1.9572e+2 (3.09e+1) + | 2.9757e+2 (4.07e+1) - | 2.5304e+2 (3.40e+1) = | 2.5177e+2 (3.90e+1) |
|  | 50 | 1.2029e+3 (5.78e+1) - | 1.1786e+3 (6.02e+1) - | 1.2007e+3 (6.85e+1) - | 1.2028e+3 (5.17e+1) - | 9.2159e+2 (6.21e+1) + | 1.0601e+3 (7.56e+1) |
|  | 100 | 2.5830e+3 (1.02e+2) = | 2.6066e+3 (8.88e+1) = | 2.5773e+3 (9.33e+1) = | 2.5892e+3 (9.36e+1) = | 2.0772e+3 (1.32e+2) + | 2.6183e+3 (1.52e+2) |
| DTLZ2 | 20 | 4.0002e-1 (6.05e-2) - | 7.9966e-1 (5.05e-2) - | 4.0522e-1 (6.35e-2) - | 7.1080e-1 (8.37e-2) - | 7.3615e-1 (9.12e-2) - | 1.3327e-1 (2.30e-2) |
|  | 50 | 2.7258e+0 (1.24e-1) - | 2.7216e+0 (1.48e-1) - | 2.7218e+0 (1.04e-1) - | 2.7282e+0 (9.89e-2) - | 1.8295e+0 (2.90e-1) + | 2.2113e+0 (3.00e-1) |
|  | 100 | 6.1119e+0 (1.75e-1) - | 6.0770e+0 (2.14e-1) - | 6.0992e+0 (1.69e-1) - | 6.0231e+0 (2.39e-1) - | 3.7760e+0 (6.32e-1) + | 5.5155e+0 (3.71e-1) |
| DTLZ3 | 20 | 8.1850e+2 (9.30e+1) = | 1.0593e+3 (9.83e+1) - | 5.4428e+2 (9.12e+1) + | 8.0779e+2 (1.27e+2) = | 7.5407e+2 (8.48e+1) = | 7.8757e+2 (1.21e+2) |
|  | 50 | 3.7581e+3 (1.54e+2) - | 3.7402e+3 (1.49e+2) - | 3.7792e+3 (1.60e+2) - | 3.6825e+3 (1.81e+2) - | 2.7113e+3 (2.10e+2) + | 3.2691e+3 (2.82e+2) |
|  | 100 | 8.3160e+3 (2.44e+2) - | 8.3178e+3 (2.26e+2) - | 8.3788e+3 (2.56e+2) - | 8.4012e+3 (1.70e+2) - | 6.0293e+3 (5.49e+2) + | 7.5396e+3 (4.07e+2) |
| DTLZ4 | 20 | 6.4549e-1 (1.51e-1) = | 6.1200e-1 (1.01e-1) = | 4.6839e-1 (7.64e-2) + | 8.5581e-1 (1.61e-1) - | 1.0335e+0 (9.79e-2) - | 6.7116e-1 (2.04e-1) |
|  | 50 | 3.0824e+0 (1.32e-1) - | 3.0912e+0 (1.38e-1) - | 3.0541e+0 (1.55e-1) - | 3.0600e+0 (1.32e-1) - | 2.2208e+0 (2.12e-1) - | 1.3388e+0 (3.75e-1) |
|  | 100 | 6.3365e+0 (2.49e-1) - | 6.4025e+0 (2.11e-1) - | 6.2863e+0 (3.06e-1) - | 6.3834e+0 (1.96e-1) - | 4.1942e+0 (4.88e-1) + | 4.8236e+0 (7.55e-1) |
| DTLZ5 | 20 | 2.9552e-1 (5.37e-2) - | 6.6141e-1 (7.89e-2) - | 3.6599e-1 (7.59e-2) - | 5.3147e-1 (1.07e-1) - | 6.2069e-1 (6.88e-2) - | 4.4066e-2 (9.38e-3) |
|  | 50 | 2.6487e+0 (1.26e-1) - | 2.6156e+0 (1.60e-1) - | 2.6170e+0 (1.74e-1) - | 2.6496e+0 (1.45e-1) - | 1.8467e+0 (2.85e-1) + | 2.0275e+0 (3.63e-1) |
|  | 100 | 6.0184e+0 (1.66e-1) - | 6.0204e+0 (2.15e-1) - | 6.0201e+0 (2.07e-1) - | 6.0227e+0 (2.01e-1) - | 3.8186e+0 (4.59e-1) + | 5.6247e+0 (3.05e-1) |
| DTLZ6 | 20 | 1.0195e+1 (6.67e-1) = | 1.4022e+1 (5.08e-1) - | 1.3657e+1 (9.13e-1) - | 8.8036e+0 (6.44e-1) + | 1.0103e+1 (8.52e-1) = | 1.0110e+1 (8.05e-1) |
|  | 50 | 4.1888e+1 (2.54e-1) - | 4.1857e+1 (2.92e-1) - | 4.1934e+1 (2.04e-1) - | 4.1936e+1 (2.07e-1) - | 3.0714e+1 (1.61e+0) + | 3.9431e+1 (1.05e+0) |
|  | 100 | 8.6484e+1 (2.20e-1) - | 8.6423e+1 (3.17e-1) - | 8.6414e+1 (3.16e-1) - | 8.6402e+1 (2.84e-1) - | 6.7583e+1 (2.25e+0) + | 8.3949e+1 (9.41e-1) |
| DTLZ7 | 20 | 3.7731e-1 (2.94e-1) + | 1.9257e+0 (5.97e-1) - | 2.6569e+0 (8.50e-1) - | 1.2734e-1 (1.55e-2) + | 6.8453e+0 (6.46e-1) - | 4.2988e-1 (1.24e-1) |
|  | 50 | 9.3703e+0 (4.80e-1) - | 9.3293e+0 (5.33e-1) - | 9.3149e+0 (4.46e-1) - | 9.2606e+0 (5.87e-1) - | 8.8796e+0 (6.29e-1) - | 1.6966e+0 (4.11e-1) |
|  | 100 | 9.9103e+0 (3.67e-1) - | 9.9875e+0 (3.17e-1) - | 9.8958e+0 (3.93e-1) - | 9.9131e+0 (3.82e-1) - | 9.9689e+0 (3.44e-1) - | 4.0322e+0 (6.86e-1) |
| WFG1 | 20 | 1.8094e+0 (1.43e-1) = | 1.9369e+0 (6.79e-2) - | 1.5963e+0 (5.29e-2) + | 1.7229e+0 (1.16e-1) = | 2.2636e+0 (6.43e-2) - | 1.7629e+0 (6.87e-2) |
|  | 50 | 2.2758e+0 (4.46e-2) - | 2.2805e+0 (3.93e-2) - | 2.2674e+0 (4.11e-2) - | 2.2859e+0 (3.90e-2) - | 2.2601e+0 (3.48e-2) - | 1.7935e+0 (1.01e-1) |
|  | 100 | 2.2380e+0 (3.72e-2) - | 2.2309e+0 (3.41e-2) - | 2.2379e+0 (3.62e-2) - | 2.2315e+0 (4.73e-2) - | 2.2769e+0 (4.67e-2) - | 1.7774e+0 (8.19e-2) |
| WFG2 | 20 | 5.0378e-1 (5.86e-2) + | 7.1282e-1 (3.00e-2) - | 5.4088e-1 (3.62e-2) + | 5.6029e-1 (7.51e-2) + | 7.6319e-1 (3.07e-2) - | 6.6273e-1 (5.28e-2) |
|  | 50 | 8.3598e-1 (2.21e-2) - | 8.5112e-1 (2.91e-2) - | 8.3881e-1 (3.02e-2) - | 8.4427e-1 (3.15e-2) - | 8.2827e-1 (2.75e-2) - | 7.3670e-1 (2.60e-2) |
|  | 100 | 8.0706e-1 (1.14e-2) - | 8.0936e-1 (1.13e-2) - | 8.1267e-1 (9.61e-3) - | 8.1026e-1 (1.09e-2) - | 8.3769e-1 (2.67e-2) - | 7.8367e-1 (2.30e-2) |
| WFG3 | 20 | 5.4063e-1 (5.63e-2) + | 7.1351e-1 (1.97e-2) - | 6.0480e-1 (3.73e-2) = | 6.8801e-1 (2.70e-2) - | 7.1916e-1 (3.33e-2) - | 5.8244e-1 (5.60e-2) |
|  | 50 | 7.7547e-1 (1.23e-2) = | 7.7245e-1 (1.01e-2) = | 7.7339e-1 (1.10e-2) = | 7.7651e-1 (1.04e-2) = | 8.2931e-1 (3.51e-2) - | 7.7597e-1 (1.94e-2) |
|  | 100 | 8.0157e-1 (5.90e-3) + | 8.0021e-1 (6.77e-3) + | 7.9862e-1 (4.98e-3) + | 8.0002e-1 (6.85e-3) + | 8.6012e-1 (3.15e-2) - | 8.3176e-1 (1.20e-2) |
| WFG4 | 20 | 4.4329e-1 (1.63e-2) - | 5.2159e-1 (9.38e-3) - | 4.6279e-1 (2.48e-2) - | 5.1135e-1 (1.24e-2) - | 5.6234e-1 (2.59e-2) - | 4.2443e-1 (3.26e-2) |
|  | 50 | 6.1301e-1 (2.94e-2) - | 6.1356e-1 (2.02e-2) - | 6.0750e-1 (2.12e-2) - | 6.1264e-1 (2.18e-2) - | 6.0295e-1 (1.41e-2) - | 5.1029e-1 (2.34e-2) |
|  | 100 | 5.7572e-1 (1.82e-2) - | 5.7868e-1 (1.76e-2) - | 5.7843e-1 (2.12e-2) - | 5.7732e-1 (1.94e-2) - | 6.1809e-1 (1.24e-2) - | 5.4974e-1 (2.36e-2) |
| WFG5 | 20 | 5.6983e-1 (4.15e-2) - | 6.4276e-1 (1.75e-2) - | 5.4967e-1 (2.79e-2) - | 4.1649e-1 (3.03e-2) + | 5.8139e-1 (1.57e-2) - | 4.7885e-1 (4.24e-2) |
|  | 50 | 7.5105e-1 (5.49e-3) - | 7.5193e-1 (6.84e-3) - | 7.5323e-1 (6.27e-3) - | 7.5295e-1 (6.42e-3) - | 6.5096e-1 (1.70e-2) = | 6.4167e-1 (3.18e-2) |
|  | 100 | 7.5057e-1 (3.40e-3) - | 7.4876e-1 (3.40e-3) - | 7.5095e-1 (3.93e-3) - | 7.4995e-1 (4.16e-3) - | 6.7921e-1 (1.44e-2) + | 7.1847e-1 (2.13e-2) |
| WFG6 | 20 | 7.7200e-1 (3.46e-2) - | 8.6526e-1 (1.61e-2) - | 7.4251e-1 (3.11e-2) - | 7.2559e-1 (3.23e-2) = | 8.9495e-1 (2.28e-2) - | 7.0700e-1 (7.22e-2) |
|  | 50 | 9.2650e-1 (7.94e-3) - | 9.2427e-1 (9.03e-3) - | 9.2745e-1 (9.85e-3) - | 9.2604e-1 (8.70e-3) - | 9.9387e-1 (1.56e-2) - | 8.7953e-1 (3.62e-2) |
|  | 100 | 9.2946e-1 (5.37e-3) - | 9.3005e-1 (5.05e-3) - | 9.2947e-1 (5.54e-3) - | 9.2974e-1 (5.16e-3) - | 1.0218e+0 (1.79e-2) - | 9.2037e-1 (2.03e-2) |
| WFG7 | 20 | 5.9714e-1 (3.32e-2) - | 6.6899e-1 (9.95e-3) - | 6.0777e-1 (2.83e-2) - | 6.7845e-1 (1.11e-2) - | 6.7568e-1 (1.75e-2) - | 5.7262e-1 (3.48e-2) |
|  | 50 | 7.0237e-1 (6.41e-3) - | 7.0570e-1 (1.07e-2) - | 7.0678e-1 (6.59e-3) - | 7.0618e-1 (5.54e-3) - | 7.3997e-1 (1.75e-2) - | 6.6520e-1 (2.27e-2) |
|  | 100 | 6.9636e-1 (3.59e-3) + | 6.9775e-1 (4.42e-3) + | 6.9714e-1 (5.28e-3) + | 6.9765e-1 (4.16e-3) + | 7.7628e-1 (1.70e-2) - | 7.0668e-1 (1.23e-2) |
| WFG8 | 20 | 5.7932e-1 (3.32e-2) + | 7.5551e-1 (1.71e-2) - | 7.0357e-1 (4.29e-2) - | 6.9293e-1 (1.74e-2) = | 7.8622e-1 (2.27e-2) - | 6.7393e-1 (4.91e-2) |
|  | 50 | 8.0295e-1 (1.39e-2) - | 8.0155e-1 (1.25e-2) - | 8.0597e-1 (1.26e-2) - | 8.0516e-1 (1.17e-2) - | 8.2493e-1 (2.10e-2) - | 7.2927e-1 (1.99e-2) |
|  | 100 | 7.6176e-1 (6.87e-3) = | 7.6296e-1 (6.79e-3) = | 7.6371e-1 (8.18e-3) = | 7.6122e-1 (6.33e-3) = | 8.3489e-1 (1.24e-2) - | 7.6667e-1 (2.33e-2) |
| WFG9 | 20 | 7.3650e-1 (5.67e-2) - | 8.5163e-1 (3.59e-2) - | 7.3331e-1 (6.35e-2) - | 8.0837e-1 (4.72e-2) - | 7.9995e-1 (3.84e-2) - | 6.3345e-1 (1.04e-1) |
|  | 50 | 9.6449e-1 (2.01e-2) - | 9.5729e-1 (1.87e-2) - | 9.6676e-1 (2.14e-2) - | 9.6549e-1 (2.39e-2) - | 8.9837e-1 (4.79e-2) - | 8.4775e-1 (6.41e-2) |
|  | 100 | 9.6627e-1 (8.48e-3) - | 9.6242e-1 (7.94e-3) - | 9.6356e-1 (8.89e-3) - | 9.6952e-1 (1.07e-2) - | 9.7352e-1 (4.25e-2) - | 9.3326e-1 (4.67e-2) |
| +/-/= | | 6/35/7 | 2/42/4 | 7/37/4 | 5/35/7 | 12/32/4 |  |

**Supplementary Table 3.** Statistical results for IGD+ values obtained by TSDEA(APD), TSDEA(CD), and TSDEA for WFG test problems. The best results are highlighted in blue.

| Problem | TSDEA(APD) | TSDEA(CD) | TSDEA |
| --- | --- | --- | --- |
| WFG1 | 1.7303e+0 (8.18e-2) - | 1.7590e+0 (7.97e-2) = | 1.7045e+0 (5.41e-2) |
| WFG2 | 7.3378e-1 (3.76e-2) = | 7.4423e-1 (2.14e-2) = | 7.1664e-1 (3.00e-2) |
| WFG3 | 7.7778e-1 (1.83e-2) = | 7.8960e-1 (1.84e-2) - | 7.7608e-1 (2.07e-2) |
| WFG4 | 4.7179e-1 (3.36e-2) = | 4.7271e-1 (2.28e-2) = | 4.6740e-1 (2.37e-2) |
| WFG5 | 5.8463e-1 (3.06e-2) = | 5.9156e-1 (3.53e-2) - | 5.6630e-1 (3.37e-2) |
| WFG6 | 8.0407e-1 (3.96e-2) - | 8.1191-1 (3.06e-2) - | 7.8550e-1 (3.60e-2) |
| WFG7 | 6.2795e-1 (2.88e-2) = | 6.3400e-1 (2.51e-2) = | 6.1482e-1 (3.28e-2) |
| WFG8 | 6.7618e-1 (3.37e-2) - | 6.8209e-1 (3.65e-2) - | 6.5611e-1 (3.13e-2) |
| WFG9 | 8.0678e-1 (8.04e-2) = | 8.1993e-1 (8.65e-2) = | 8.0587e-1 (7.22e-2) |
| +/-/= | 0/3/6 | 0/4/5 |  |

**Supplementary Table 4.** Statistical results for IGD values obtained by TSDEA(D), TSDEA(A), and TSDEA for DTLZ test problems. The best results are highlighted in blue.

| Problem | TSDEA(D) | TSDEA(A) | TSDEA |
| --- | --- | --- | --- |
| DTLZ1 | 8.9755e+1 (2.14e+1) - | 8.1046e+1 (2.22e+1) = | 7.5724e+1 (2.00e+1) |
| DTLZ2 | 5.1911e-2 (2.83e-3) - | 1.7671e-1 (1.94e-2) - | 4.9966e-2 (3.17e-3) |
| DTLZ3 | 2.2739e+2 (3.62e+1) - | 2.0911e+2 (5.01e+1) = | 1.9560e+2 (4.55e+1) |
| DTLZ4 | 4.4434e-1 (1.97e-1) - | 3.2843e-1 (4.74e-2) = | 3.9051e-1 (1.94e-1) |
| DTLZ5 | 1.4513e-2 (2.73e-3) - | 2.0671e-1 (3.70e-2) - | 9.6892e-3 (8.58e-4) |
| DTLZ6 | 3.1754e+0 (4.92e-1) - | 5.3729e+0 (4.47e-1) - | 2.2166e+0 (4.47e-1) |
| DTLZ7 | 2.7589e-1 (2.04e-1) - | 1.1073e+0 (3.76e-1) - | 1.3229e-1 (1.01e-1) |
| +/-/= | 0/7/0 | 1/4/2 |  |
